# Population Genetic Differentiation and Evolutionary History in *Liriodendron* Revealed by Stress‐Related Single‐Copy Genes

**DOI:** 10.1002/ece3.72182

**Published:** 2025-09-22

**Authors:** Yanli Cheng, Heyang Yuan, Lichun Yang, Xi Chen, Huogen Li

**Affiliations:** ^1^ State Key Laboratory of Tree Genetics and Breeding, Co‐Innovation Center for Sustainable Forestry in Southern China Nanjing Forestry University Nanjing Jiangsu China; ^2^ College of Architecture Anhui Science and Technology University Bengbu Anhui China; ^3^ Anhui Hongsen High‐Tech Forestry Co., Ltd. Guoyang Anhui China

**Keywords:** *Liriodendron*, nucleotide variation, population history, population origin, stress‐related single‐copy genes

## Abstract

*Liriodendron* was widely distributed across the Northern Hemisphere during the early to middle Miocene, but now is confined to East Asia and North America. As a relic plant of environmental shifts, *Liriodendron* provides valuable insights into the adaptation of temperate trees to changing environments. To investigate the genetic differentiation and evolutionary history of *Liriodendron*, 8667 single‐copy orthologs were identified as ideal markers owing to their slow mutation rate and generational stability. Their most enriched functions, specifically in energy metabolism, photosynthetic efficiency, and stress resilience, underpinned *Liriodendron'*s survival in harsh conditions. Three single‐copy genes (*LtDHN2*, *LtDHN3*, and *LtTLP11*) associated with abiotic stress tolerance were cloned and sequenced in 20 *Liriodendron* populations. *LtDHN2*, *LtDHN3*, and *LtTLP11* exhibited high SNP density (1/15.80–1/19.45 bp) and significant interspecific differentiation (*F*_st_ = 0.71–0.94), whereas intraspecific variation was minimal, suggesting recent expansion. Both *L. tulipifera* and the eastern and western populations of *L. chinense* served as ancestral populations, exhibiting asymmetric historical gene flow among these populations. Divergence events were driven by disrupted trans‐Beringian gene flow (11.4–13.4 Ma) and Quaternary glaciations (2.19–2.23 Ma). The effective population size of *L. chinense* underwent rapid expansion during the Early Last Glacial and the Last Interglacial, while *L. tulipifera* exhibited recolonization during the warmer MIS 3 interstadial resilience phase, the fluctuating substages of the Last Interglacial, and the late Penultimate Glacial Maximum warming phase. These findings highlight how paleogeographic constraints, climatic oscillations, and locus‐specific selection have shaped the evolutionary trajectory and current disjunct distribution of *Liriodendron*.

## Graphical abstract

Identification of single‐copy ortholog genes and genetic differences in *Liriodendron*: In *Liriodendron*, 8667 single‐copy ortholog genes were identified, including three stress‐related genes (*LtDHN2*, *LtDHN3*, and *LtTLP11*) that were cloned and amplified across multiple populations. Genetic polymorphism analysis revealed significant variation between *L. chinense* and *L. tulipifera* , moderate variation within populations, and lesser variation within species among populations. Ancestral population analysis: *L. tulipifera* and the eastern and western populations of *L. chinense* all served as ancestral populations of the *Liriodendron* genus. The divergence between *L. chinense* and *L. tulipifera* is estimated to have occurred approximately 11.4–13.4 million years ago. Both the eastern and western populations of *L. chinense* trace their ancestry back to each other's ancestral populations, dating back to around 2.19–2.23 million years ago. The effective population size of *L. chinense* underwent rapid expansion during the Early Last Glacial and the last interglacial period (*LtTLP11*: 0.05–0.13 Ma), while *L. tulipifera* exhibited recolonization during the warmer MIS 3 interstadial resilience phase, the fluctuating substages of the Last Interglacial, and the late Penultimate Glacial Maximum warming phase (*LtDHN2*: 0.025–0.06 Ma, *LtTLP11*: 0.07–0.15 Ma).

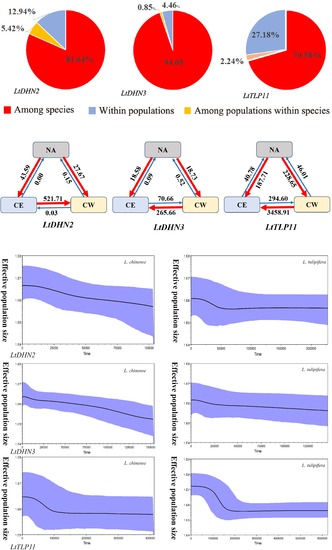

## 1 Introduction

During the Cretaceous period, the plant kingdom changed significantly. Angiosperms emerged gradually, developed rapidly, and became dominant by the late Cretaceous, replacing gymnosperms as the main class of terrestrial plants (Benton et al. 2022). Parks et al. (1983) demonstrated that *Liriodendron* Linnaeus of the Magnoliaceae family was widely present during this period. Genome‐based studies reveal that magnoliids are sister to the clade consisting of eudicots and monocots. *Liriodendron* is an early diverging basal lineage within magnoliids (Chen et al. 2018; Hu, Gong, et al. 2025; Hu, Wang, et al. 2025). Molecular dating indicates that the *Liriodendron* lineage diverged from core Magnoliid approximately 93.5 million years (Ma) ago, during the Cenomanian (Nie et al. 2008). These findings underscore the necessity of further investigating the evolutionary origins and history of *Liriodendron* to understand angiosperm phylogeny.

Genomic evidence indicates that a *Liriodendron* whole‐genome duplication (WGD) event occurred approximately 116 Ma, with a synonymous substitution rate of 3.02 × 10^−9^ substitutions per synonymous site per year (Chen et al. 2018). This WGD likely predated the divergence of Magnoliaceae and Lauraceae. Pollen records of *Liriodendron* were found in Early Cretaceous, Aptian–Albian strata at Baihedong, Guangzhou, China (Yang 2021). Importantly, this represents the oldest record of *Liriodendron*, suggesting southern China to be its likely origin (Shen, Tu, et al. 2022; Shen, Xia, et al. 2022). The earliest macrofossils of *Liriodendron* have been documented in the Cenomanian strata from the Late Cretaceous of northeastern Virginia, USA (Maryland Geological Survey 1916). Subsequently, Late Cretaceous fossils of this genus were found in Kustanay, Kazakhstan; Honshu, Japan; Eschborn and Quedlinburg, Germany; North Carolina, USA; and Vancouver, Canada (Bell 1957; Frumin and Friis 1996). Despite abundant Cretaceous fossils, no clear hypothesis exists regarding the origin and geographic centers of *Liriodendron*.

By the beginning of the Tertiary, the angiosperms that evolved during the Cretaceous had become the main community (Ding et al. 2025). Fossil evidence indicates that *Liriodendron* spread widely across the Northern Hemisphere during the Late Cretaceous and Tertiary, alongside the rise of angiosperms (Fetter 2014). Early Tertiary fossils of *Liriodendron* were found in the strata of Greenland, Canada, Iceland, Siberia, Colorado, and Oregon in the USA (Cevallos‐Ferriz and Stockey 1990; Taylor 1990). Moreover, a large number of *Liriodendron* fossils were found in late Tertiary strata across regions such as the Czech Republic, Japan, Korea, Central Asia, Europe, the Pacific Northwest, the USA, China, Siberia, and Italy (Geissert et al. 1990). As the Atlantic Ocean covered the land bridge between Europe and North America, gene flow ceased among *Liriodendron* populations in North America, East Asia, and Europe. Fossil data indicated that temperate plants, including *Liriodendron*, could no longer survive in the Bering region during the late Miocene. This suggested that gene exchange of *Liriodendron* populations between North America and East Asia through the Bering Land Bridge was interrupted, leading to their disjunct East Asian–North American distribution (Parks and Wendel 1990).

*L. chinense* and *L. tulipifera* have a characteristic disjunct distribution across East Asia and North America. While morphologically similar, *L. tulipifera*, with its broad distribution and abundant genetic resources, exhibits strong adaptability compared to *L. chinense* (Nezu et al. 2022; Quassinti et al. 2019). *L. chinense* is a rare relict species found in the mountainous regions to the south of the Yangtze River basin (22° N–33° N, 103° E–120° E), at *elevations* of 700–1900 m. Hence, its distribution is characterized by a “one belt and six islands” pattern, indicating significant barriers to range expansion (Shen et al. 2019). Due to threats, including small population sizes, low natural seed germination rates, harsh environments, and human interference, *L. chinense* has been designated as a National Category II Protected precious species in the Information System of Chinese Rare and Endangered Plants (ISCREP) and listed as Near Threatened on the IUCN Red List of Threatened Species (Phan 2015). In contrast, *L. tulipifera* thrives as a dominant pioneer species in North America, with a robust presence in the eastern United States and southern Canada (27° N–42° N, 77° W–94° W) and primarily occurring below 300 m in elevation. It has a continuous distribution in the Appalachian Mountains, extending from Pennsylvania to Georgia (LeBlanc et al. 2020). Although the current distribution surveys of *L. chinense* and *L. tulipifera* are well defined, there has been little investigation into the historical population sizes of this genus and the evolutionary dynamics that have unfolded over time.

Single‐copy orthologous genes exhibit a lower mutation rate, enabling them to remain relatively stable across generations and accurately reflect genetic diversity and evolutionary history. These genes, inherited from both parents, are minimally influenced by recombination and retain parental alleles for extended periods, making them rich information sites (Nakandala et al. 2023). Compared to cpDNA, single‐copy gene segments provide a broader depiction of the effective population size, which is particularly pertinent for small natural populations, such as the endangered *L. chinense* (Nekola et al. 2023). Widely utilized as phylogenomic markers in systematics, these genes offer clear resolution across various taxonomic levels and are employed to study geographic distribution patterns, phylogenetic relationships, and population history dynamics among closely related species (Sun et al. 2020). Naranjo et al. (2024) indicated that despite the challenges posed by polyploidy, single‐copy genes provided reliable phylogenetic signals for inferring relationships among polyploid taxa. Dong et al. (2019) demonstrated that single‐copy nuclear loci developed from multiple transcriptomes offered high‐resolution phylogenetic insights into Chinese and Vietnamese scaly ferns.

Stress‐related single‐copy genes, particularly those that confer adaptive functions in response to low temperatures, water scarcity, salt stress, and biotic challenges exacerbated by climate change, are critical for the adaptability of *Liriodendron* (Chaudhry and Sidhu 2022). Among the key genetic players, dehydrins (DHNs) and thaumatin‐like proteins (TLPs) have emerged as functionally conserved yet ecologically versatile candidates, whose roles in climate adaptation warrant in‐depth exploration (Feng et al. 2024; Szlachtowska and Rurek 2023). Dehydrins, intrinsically disordered proteins, exhibit exceptional hydrophilicity and structural flexibility, enabling them to stabilize cellular membranes and protect macromolecules under dehydration conditions caused by drought or freezing (Zuo et al. 2023). Their upregulation during abiotic stress correlates with enhanced osmotic adjustment and redox homeostasis, suggesting an evolutionary adaptation to fluctuating water availability. Similarly, thaumatin‐like proteins (TLPs) defend against fungal pathogens through hydrolytic activity and mitigate osmotic and ionic imbalances during salt stress (Hu, Gong, et al. 2025; Hu, Wang, et al. 2025). The prevalence of TLPs across diverse plant lineages implies their adaptive significance in environments where biotic and abiotic stressors co‐occur (De Vaz Sousa et al. 2024). Our research harnesses molecular phylogeographic theories and methodologies, utilizing sequence variation from three stress‐related single‐copy genes across three genotypes of each *Liriodendron* population to infer the population genetic differentiation and evolutionary history of the genus.

## 2 Material and Methods

### 2.1 Plant Materials of *Liriodendron*

The plant materials used in this study consisted of 20 natural populations of *Liriodendron*, including 14 populations of *L. chinense* and 6 populations of *L. tulipifera* . At least three adult individuals were collected from each population. All samples were obtained from the provenance trial forest of *Liriodendron* at the Xiashu Experimental Forest Farm of Nanjing Forestry University and natural populations of *Liriodendron*. Fresh leaves or buds from each individual were collected and dried in silica gel for preservation. Detailed information on the sources of the sampling is provided in Table 1.

**TABLE 1:** Sample sites and number of individuals of 20 *Liriodendron* populations.

Group	Site	Longitude	Latitude	Elevation (m)	Sample size
CE	Anji, Zhejiang, CHN (AJ)	119.43° E	30.4° N	935	3
	Songyang, Zhejiang, CHN (SY)	119.6° E	28.5° N	138	3
	Huangshan, Anhui, CHN (HS)	116.1° E	30.17° N	1250	3
	Lushan, Jiangxi, CHN (LS)	116° E	29.53° N	1167	3
	Wuyishan, Fujian, CHN (WYS)	117.76° E	27.84° N	1700	3
CW	Xianning, Hubei, CHN (XN)	114.2° E	29.8° N	68	3
	Exi, Hubei, CHN (EX)	109° E	30.3° N	1180	3
	Suining, Hunan, CHN (SN)	110.2° E	26.33° N	1500	3
	Maoershan, Guangxi, CHN (MES)	110.4° E	25.87° N	1100	3
	Yinjiang, Guizhou, CHN (YJ)	108.61° E	27.89° N	1560	3
	Songtao, Guizhou, CHN (ST)	109.32° E	28.16° N	903	3
	Liping, Guizhou, CHN (LP)	109.19° E	26.34° N	421	3
	Youyang, Sichuan, CHN (YY)	108.8° E	28.82° N	890	3
	Yuanyang, Yunnan, CHN (YUY)	103.07° E	23.03° N	1540	3
NA	Pennsyjvania, USA (PE)	77.01° W	40.26° N	150	3
	North Carolina, USA (NC)	77.15° W	36.07° N	8	3
	Missouri, USA (MI)	92.23° W	38.87° N	245	3
	Georgia, USA (GE)	84.76° W	34.64° N	230	3
	South Carolina, USA (SC)	80.82° W	33.83° N	65	3
	Louisiana, USA (LO)	91.02° W	30.42° N	18	3

Abbreviations: CE, *L. chinense* populations from Eastern China; CW, *L. chinense* populations from Western China; NA, *L. tulipifera* populations from North America.

### 2.2 Identification of Single‐Copy Genes in *Liriodendron*

The amino acid FASTA files of all gene coding sequences for *L. tulipifera* and *L. chinense* were downloaded from the Phytozome (https://phytozome‐next.jgi.doe.gov/) and NCBI (https://www.ncbi.nlm.nih.gov/datasets/genome/) databases, respectively. Single‐copy ortholog sequences were identified and compared using OrthoFinder v2.5.5. All identified single‐copy ortholog sequences were merged into one file and then separated into sequences specific to *L. chinense* and *L. tulipifera* . Functional annotation of *L. tulipifera* single‐copy orthologs was performed using the online tool eggNOG‐mapper (http://eggnog‐mapper.embl.de/). Finally, the annotation results were analyzed using Go (Gene Ontology) and KEGG (Kyoto Encyclopedia of Genes and Genomes) enrichment analyses and Enrichment Bar Plot tools available in TBtools‐II (Chen et al. 2023).

### 2.3 Cloning and Sequencing of Stress‐Related Single‐Copy Genes in *Liriodendron*

Given that *L. chinense* exhibits notably inferior adaptability to cold and drought stress in comparison to *L. tulipifera* , three single‐copy genes were meticulously chosen for further study. These genes include *LtDHN2* and *LtDHN3*, which are dehydrins known to participate in the osmotic stress response, as well as *LtTLP11*, a thaumatin‐like protein linked to both pathogen defense and abiotic stress resilience. Total RNA and DNA were extracted from *Liriodendron* plants using the RNAprep Pure Plant Kit and Plant Genomic DNA Kit (Tiangen Biotech, China). The first strand of cDNA, 3′RACE, and 5′RACE cDNA were synthesized using the RevertAid strand cDNA Synthesis Kit (Thermo Fisher Scientific), the 3′‐Full RACE Core Set with PrimeScript RTase and the 5′Full RACE Kit (Takara, Japan), respectively. The intermediate fragment, RACE and ORF primers were designed using Oligo7 software (Table S1). A total of 5 μL 10 × LA PCR Buffer II (Mg^2+^ Free), 5 μL MgCl_2_ (25 mmol/L), 8 μL dNTP Mixture (2.5 mmol/L each), 2 μL upstream/downstream primers (2.5 mmol/L each), 0.5 μL TaKaRa LA Taq (5 U/μL), 2 μL cDNA, and 25.5 μL ddH_2_O were used in a 50 μL RACE PCR system. The PCR amplification program was: 94°C for 3 min; 35 cycles of 94°C for 30 s, annealing temperature for 30 s, and 72°C for 1 min; 72°C for 10 min; and 4°C storage. Each gene was verified using ORF finder (https://www.ncbi.nlm.nih.gov/orffinder/) and confirmed by sequencing. Subsequently, 120 *Liriodendron* individuals were sequenced. The 50 μL PCR system contained 25 μL 2 × TransStart FastPfu PCR SuperMix, 2 μL ORF upstream/downstream primers (2.5 mmol/L each), 2 μL cDNA, and 19 μL ddH_2_O. The corresponding PCR program was set as follows: 94°C for 3 min; 94°C for 20 s, 50°C for 20 s, 72°C for 1 min, 35 cycles; 72°C for 10 min; and 4°C for storage. All PCR products were separated and recovered by agarose gel electrophoresis, then ligated to the pEASY‐T1 vector (Transgen Biotech, China). The recombinant plasmids were transformed into *Escherichia coli* competent cells. Transformants were cultured and screened, and positive clones were sequenced by Genscript (China).

### 2.4 Genetic Diversity and Sequence Variation in *Liriodendron*

*S* (the polymorphic sites), Indels (insertion–deletion sites), *π* (nucleotide diversity), and *θw* (theta [per site] from *S*) of *Liriodendron* populations were calculated using DnaSP v5 and MEGA v12 (Kumar et al. 2024; Librado and Rozas 2009). The genetic variation and *F*_st_ (Fixation indices) of the *Liriodendron* populations were calculated at three levels of variation (among species, among populations within species, and within populations) using an AMOVA (Analysis of Molecular Variance) in Arlequin v3.5.2.2 (Excoffier and Lischer 2010).

### 2.5 Inference of Population Origin and Divergence Time in *Liriodendron*

**FIGURE 1:**
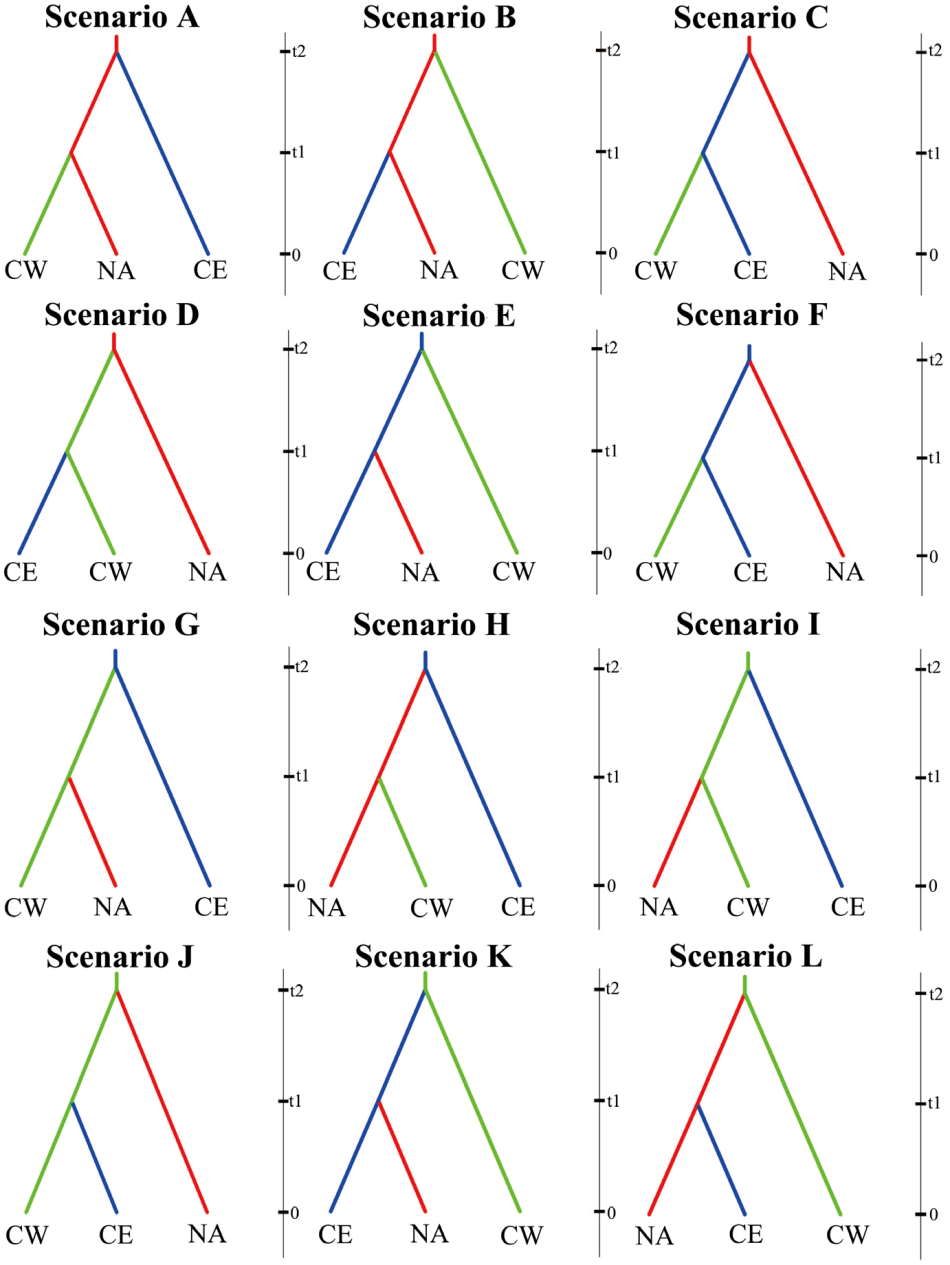
Graphical representation of putative demographic scenarios tested to explain the current distribution of *Liriodendron*. CE, *Liriodendron chinense* populations from Eastern China; CW, *Liriodendron chinense* populations from Western China; NA, *Liriodendron tulipifera* populations from North America.

Past demographic events in *Liriodendron* populations were inferred using DIYABC v2.1.0 (Cornuet et al. 2008). Although STRUCTURE analysis defined the eastern (CE) and western (CW) populations of *L. chinense* (typically 3‐lobed leaves) and *L. tulipifera* (NA; typically 5‐lobed leaves) as divergent groups, the occasional appearance of swapped morphologies between them suggests an evolutionary connection (Shen, Tu, et al. 2022; Shen, Xia, et al. 2022). The early fossil record of *Liriodendron* supports the hypothesis that angiosperms originated in the tropics of Asia, the Americas, and Africa (Friis et al. 2025; Retallack and Dilcher 1986). Hence, 12 hypothesized evolutionary scenarios are summarized (Figure 1).

Uniform priors for the scaled effective population sizes were set, ranging from 219,000 to 326,000 for CE and CW and from 800,000 to 900,000 for NA. Parameters for time 1 and time 2 were randomly selected within the intervals of [219,000; 326,000] and [1,139,000; 1,336,000], respectively, with reference to the time period during which complete differentiation occurred. A total of 12,000,000 simulations were performed to test the 12 scenarios (Figure 1). The posterior probabilities of the scenarios were computed, and confidence in the scenario choice was evaluated to ensure the robustness of the inferred population history.

### 2.6 Estimation of Historical Gene Flow Among *Liriodendron* Populations

Historical gene flow among the populations of *Liriodendron* was estimated using Lamarc v2.1.10 (http://evolution.genetics.washington.edu/lamarc/index). This software employs coalescent theory with a maximum likelihood approach under the F84 model to estimate the direction and magnitude of historical gene flow between *L. chinense* and *L. tulipifera* and between the eastern and western populations of *L. chinense* . Parameters were set to run 50 initial chains, each possessing a length of 5000 steps, with the first 5000 steps of each chain discarded as burn‐in. The final analysis involved 10 chains, each with a length of 50,000 steps, discarding the first 5000 steps. Tracer v1.7 was then used to assess the effective sampling range of various indicators, considering computations valid if the effective sample size exceeded 200 (Rambaut et al. 2018).

### 2.7 Inferring Population History of *Liriodendron*

Two methods were employed to calculate the evolutionary rates of *LtDHN2*, *LtDHN3*, and *LtTLP1*. (1) The formula for estimating mean genetic identity was as follows: $I_{\mathrm{EA}}=\frac{1}{1+2\bar{v}t}\left[1+\sum_{r=1}^{\infty }\frac{\left(2r\right)!}{{\left(r!\right)}^{2}}{\left(\frac{\bar{v}t}{1+2\bar{v}t}\right)}^{2r}\right]$, where *I*_EA_ is the mean genetic identity; $\bar{v}$ is the average mutation rate at the site; *t* is the evolutionary time of the species, and r is the number of loci. The average mutation rate of the three adaptive genes was estimated using the functional relationship between the mean genetic identity and mutation rate. (2) Using BEAST v1.8.4 (http://beast.bio.ed.ac.uk/), the evolutionary rates of the three genes were calculated to be consistent with the divergence time of *L. chinense* and *L. tulipifera* (10–16 Ma). The HKY model was selected, and an uncorrelated relaxed exponential clock model and a constant size tree prior model were used to construct the UPGMA phylogenetic tree. The Markov chain Monte Carlo process (MCMC) was run for 10,000,000 generations, with sampling every 1000 generations, and the first 10% was discarded as burn‐in. Finally, Tracer v1.7 was used to examine the evolutionary rates of these three genes. Using the estimated average mutation rate as a prior distribution, the Bayesian Skyline Plot (BSP) was applied in BEAST v1.8.4. The standard MCMC sampling procedure was followed as previously described and the BSP results were visualized and constructed using Tracer v1.7.

## 3 Results

### 3.1 Identification and Functional Annotation of Single‐Copy Ortholog Genes in *Liriodendron*

**FIGURE 2:**
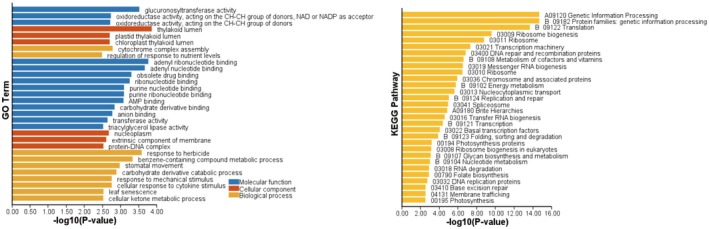
GO and KEGG term enrichments of all single‐copy genes in *Liriodendron*.

Using OrthoFinder v2.5.5, 8667 single‐copy ortholog genes were identified in *Liriodendron*, representing 15.60% of the total genes in *L. chinense* and 18.26% of the total genes in *L. tulipifera* . GO enrichment analysis of the single‐copy genes was conducted, and the top two enriched GO terms in the three GO categories (molecular function [MF], cellular component [CC], biological processes [BP]) were identified as adenyl ribonucleotide binding, adenyl nucleotide binding; thylakoid lumen, plastid thylakoid lumen; response to herbicide, and benzene‐containing compound metabolic process (Figure 2). The top three enriched KEGG categories were genetic information processing, protein families: genetic information processing, and translation (Figure 2).

### 3.2 Cloning and Characterization of Three Stress‐Related Single‐Copy Genes in *Liriodendron*

Stress‐related single‐copy genes were selected from the single‐copy ortholog gene database for the study. Using EST sequences as references, we cloned three genes from the DHNs and TLPs families by RACE technique. The amplification results of the intermediate, 3′, 5′, and full‐length cDNA sequences are shown in Appendix A. The three genes were named *LtDHN2*, *LtDHN3*, and *LtTLP11* by identifying them in the genome and blasting against the nucleotide collection (nr/nt) database. *LtDHN2* and *LtDHN3* belong to the SK_n_ subfamily (Appendix B), and CCD server analysis reveals that they have the Dehydrin PF00257 conserved domain. *LtDHN2* protein has one S segment (QRSNSSSSSSSSDE) and four K segments (EKKKKKKKGSLKEKL, EKKEEEKKVA ETEEC, EKKGFLEKIKEKLPG, EKKGLLEKIKEKLPG). *LtDHN3* protein has one S segment (LHRSHSSSSSSSDE) and two K segments (EKKGFL EKMKEKLPG, EKKGLLEKIKEKLPG). *LtTLP11* protein has a Thaumatin family PFO0314 domain of the thaumatin‐like subfamily (Appendix B).

### 3.3 Genetic Polymorphism Analysis of Three Stress‐Related Single‐Copy Genes in *Liriodendron*

Polymorphisms and genetic variations in *LtDHN2*, *LtDHN3*, and *LtTLP11* were analyzed in 120 individuals from 20 *Liriodendron* populations. The dataset contained 193, 1480, and 63 SNPs for the three genes, respectively, comprising 84, 188, and 49 polymorphic sites, and 109, 1292, and 14 indels. The SNP occurrence frequencies for the three genes were 1 SNP per 17.35 bp, 1 SNP per 19.45 bp, and 1 SNP per 15.80 bp, respectively. The *π* and *θw* values were 0.0118, 0.0187, 0.0114 and 0.0116, 0.0148, 0.0120, respectively. The distribution of polymorphic sites of the three genes across populations is shown in Table 2. The differences in the *π* and *θw* values between *L. chinense* and *L. tulipifera* for *LtDHN2*, *LtDHN3*, and *LtTLP11* were relatively minor. Specifically, 31 SNPs (16.06% of total SNPs) in *LtDHN2*, 675 SNPs (45.61%) in *LtDHN3*, and no SNPs in *LtTLP11* exhibited complete fixation in the two species (Table 4). The differences in the *π* and *θw* values between the eastern and western populations of *L. chinense* were also minimal, with no completely fixed SNPs observed between these groups. Notably, the XN population displayed relatively high genetic diversity for all three genes.

**TABLE 2:** The nucleotide variation and diversity of *LtDHN2*, *LtDHN3*, and *LtTLP11* in *Liriodendron* populations.

Genetic parameters		Gene										
		*LtDHN2*					*LtDHN3*					*LtTLP11*
Population		Exon‐1159 bp	Intron 899 bp	Exon‐2399 bp	Total 1457 bp	5′UTR 54 bp	Exon‐1192 bp	Intron 2910 bp	Exon‐2417 bp	3′UTR 83 bp	Total 3656 bp	Total 774 bp
AJ	*S*/Indels	0/0	3/21	0/21	3/42	0/0	0/3	21/665	1/6	0/0	22/674	6/0
	*π/θw* (%)	0/0	0.18/0.15	0/0	0.11/0.09	0/0	0/0	0.39/0.41	0.08/0.11	0/0	0.30/0.32	0.26/0.34
SY	*S*/Indels	2/0	10/23	2/21	14/44	0/0	0/3	13/664	0/6	0/0	13/673	4/0
	*π*/*θw* (%)	0.42/0.55	0.38/0.50	0.23/0.23	0.34/0.43	0/0	0/0	0.28/0.25	0/0	0/0	0.21/0.19	0.23/0.23
HS	*S*/Indels	0/0	0/21	0/21	0/42	0/0	0/3	19/690	1/6	0/0	20/699	6/0
	*π/θw* (%)	0/0	0/0	0/0	0/0	0/0	0/0	0.38/0.38	0.15/0.11	0/0	0.30/0.30	0.28/0.34
LS	*S*/Indels	1/0	5/22	0/21	6/43	0/0	0/3	26/663	0/6	0/0	26/672	20/1
	*π/θw* (%)	0.21/0.28	0.21/0.25	0/0	0.16/0.19	0/0	0/0	0.79/0.50	0/0	0/0	0.37/0.38	1.29/1.13
WYS	*S*/Indels	0/0	0/20	0/6	0/26	0/0	0/3	21/665	1/6	0/0	22/674	17/0
	*π/θw* (%)	0/0	0/0	0/0	0/0	0/0	0/0	0.43/0.41	0.13/0.11	0/0	0.34/0.32	0.73/1.02
CE	*S*/Indels	2/0	14/23	2/21	18/44	0/0	0/3	47/691	1/6	0/0	48/700	29/1
	*π/θw* (%)	0.12/0.32	0.19/0.0	0.05/0.13	0.14/0.32	0/0	0/0	0.47/0.54	0.1/0.1	0/0	0.37/0.41	0.60/0.95
XN	*S*/Indels	0/0	9/21	2/6	11/27	0/0	2/3	32/712	1/6	0/0	35/721	17/0
	*π/θw* (%)	0/0	0.44/0.45	0.27/0.22	0.35/0.34	0/0	0.35/0.5	0.63/0.64	0.08/0.06	0/0	0.52/0.52	0.73/0.96
EX	*S*/Indels	0/0	4/22	1/6	5/28	0/0	0/3	19/663	0/6	0/0	19/672	1/0
	*π/θw* (%)	0/0	0.18/0.20	0.09/0.11	0.13/0.15	0/0	0/0	0.40/0.37	0/0	0/0	0.31/0.28	0.07/0.06
SN	*S*/Indels	0/0	1/21	1/21	2/42	0/0	1/3	27/709	0/6	0/0	28/718	18/0
	*π/θw* (%)	0/0	0.06/0.05	0.14/0.12	0.08/0.06	0/0	0.18/0.23	0.54/0.54	0/0	0/0	0.42/0.42	0.76/1.02
MES	*S*/Indels	0/0	5/21	2/6	7/27	0/0	1/3	18/709	0/6	0/0	19/718	17/0
	*π/θw* (%)	0/0	0.26/0.25	0.29/0.22	0.24/0.21	0/0	0.18/0.23	0.45/0.36	0/0	0/0	0.35/0.28	0.78/0.96
YJ	*S*/Indels	0/0	1/21	1/24	2/45	0/0	1/3	19/663	0/6	0/0	20/672	20/0
	*π/θw* (%)	0/0	0.04/0.05	0.09/0.12	0.1/0.1	0/0	0.18/0.23	0.34/0.37	0/0	0/0	0.27/0.29	0.90/1.13
ST	*S*/Indels	0/0	3/21	0/21	3/42	0/0	0/3	29/664	0/9	0/0	29/676	7/0
	*π/θw* (%)	0/0	0.16/0.15	0/0	0.05/0.06	0/0	0/0	0.51/0.57	0/0	0/0	0.38/0.43	0.33/0.40
LP	*S*/Indels	1/0	4/23	2/21	7/44	0/0	1/3	20/709	0/6	0/0	21/718	4/10
	*π/θw* (%)	0.21/0.28	0.18/0.20	0.25/0.23	0.20/0.22	0/0	0.28/0.23	0.49/0.40	0/0	0/0	0.38/0.31	0.20/0.23
YY	*S*/Indels	2/0	6/21	1/9	9/30	0/0	0/3	5/663	0/6	0/0	5/672	22/1
	*π/θw* (%)	0.59/0.55	0.41/0.30	0.15/0.11	0.36/0.28	0/0	0/0	0.09/0.10	0/0	0/0	0.07/0.07	0.95/1.25
YUY	*S*/Indels	0/0	10/21	1/21	11/42	0/0	1/3	17/710	0/9	0/0	18/722	1/1
	*π/θw* (%)	0/0	0.54/0.50	0.14/0.12	0.37/0.34	0/0	0.18/0.23	0.27/0.34	0/0	0/0	0.21/0.27	0.08/0.06
CW	*S*/Indels	3/0	26/23	4/24	33/47	0/0	4/3	75/712	1/9	0/0	80/724	34/12
	*π/θw* (%)	0.11/0.41	0.33/0.65	0.19/0.23	0.27/0.51	0/0	0.15/0.46	0.57/0.75	0.01/0.05	0/0	0.44/0.60	0.53/0.98
ZG	*S*/Indels	5/0	34/25	6/24	45/49	0/0	4/3	102/740	2/9	0/0	108/752	41/13
	*π/θw* (%)	0.12/0.63	0.31/0.78	0.18/0.32	0.25/0.64	0/0	0.10/0.42	0.57/94	0.04/0.10	0/0	0.44/0.74	0.55/1.07
PE	*S*/Indels	1/0	11/82	0/0	12/82	0/0	0/0	10/1067	3/3	1/0	14/1070	10/0
	*π/θw* (%)	0.34/0.28	0.70/0.59	0/0	0.46/0.38	0/0	0/0	0.27/0.24	0.39/0.32	0.64/0.53	0.27/0.24	0.49/0.57
NC	*S*/Indels	0/0	1/82	0/0	1/82	0/0	1/3	26/1066	2/3	1/0	30/1072	9/0
	*π/θw* (%)	0/0	0/0.1	0/0	0.02/0.03	0/0	0.28/0.23	0.65/0.62	0.16/0.21	0.4/0.5	0.53/0.51	0.59/0.51
MI	*S*/Indels	1/0	8/82	2/3	11/85	0/0	2/0	15/1067	1/6	2/0	20/1073	18/0
	*π/θw* (%)	0.34/0.28	0.52/0.43	0.27/0.22	0.43/0.35	0/0	0.45/0.46	0.43/0.36	0.08/0.11	1.29/1.05	0.40/0.34	0.76/1.02
GE	*S*/Indels	0/0	1/82	1/0	2/82	0/0	1/0	24/1066	1/3	1/0	27/1069	5/0
	*π/θw* (%)	0/0	0/0.1	0.08/0.11	0.05/0.06	0/0	0.17/0.23	0.60/0.57	0.08/0.11	0.40/0.53	0.50/0.46	0.22/0.28
SC	*S*/Indels	2/0	7/82	0/0	9/82	0/0	1/0	22/1069	2/3	1/0	26/1072	4/1
	*π/θw* (%)	0.67/0.55	0.46/0.38	0/0	0.35/0.29	0/0	0.28/0.23	0.66/0.52	0.16/0.21	0.40/0.53	0.53/0.44	0.17/0.23
LO	*S*/Indels	1/0	8/82	2/0	11/82	0/0	0/0	22/1123	1/3	3/0	26/1126	5/0
	*π/θw* (%)	0.34/0.28	0.52/0.43	0.27/0.22	0.43/0.35	0/0	0/0	0.60/0.54	0.08/0.11	1.45/1.58	0.49/0.45	0.24/0.28
NA	*S*/Indels	2/0	17/82	3/3	22/85	0/0	2/0	42/1128	7/6	3/0	54/1134	28/1
	*π/θw* (%)	0.33/0.31	0.49/0.50	0.16/0.18	0.37/0.39	0/0	0.29/0.25	0.60/0.57	0.20/0.41	0.78/0.87	0.50/0.52	0.53/0.87

Abbreviations: *π*, nucleotide diversity; *θw*, theta (per site) from *S*; CE, *L. chinense* populations from Eastern China; CW, *L. chinense* populations from Western China; NA, *L. tulipifera* populations from North America; *S*, SNPs sites; Indels, insertion or deletion sites; ZG, *L. chinense* populations.

### 3.4 Genetic Variation Analysis of Three Stress‐Related Single‐Copy Genes in *Liriodendron*

**FIGURE 3:**
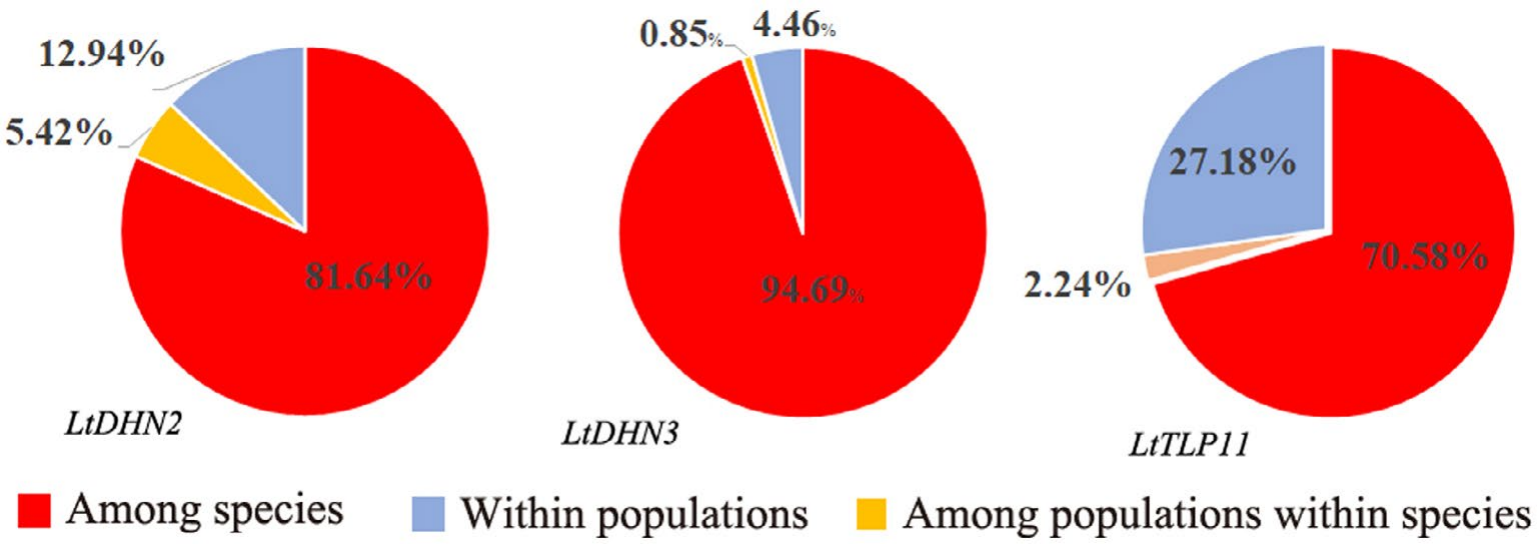
The analysis of molecular variance (AMOVA) results of *LtDHN2*, *LtDHN3*, and *LtTLP11* genes in *Liriodendron* populations.

Figure 3 indicated that the genetic variation of *LtDHN2*, *LtDHN3*, and *LtTLP11* gDNA was primarily due to differences between *L. chinense* and *L. tulipifera* , explaining 81.64%, 94.69%, and 70.58% of the total variation, respectively. The remaining variation in the three genes was mainly attributed to differences among individuals within populations, accounting for 12.94%, 4.46%, and 27.18% of the total variation. Variation among different populations within *Liriodendron* was the lowest, accounting for 5.42%, 0.85%, and 2.24% of the total variation, respectively. The *F*_st_ values between *L. chinense* and *L. tulipifera* were 0.71, 0.75, and 0.71. The *F*_st_ values between the eastern and western populations of *L. chinense* were 0.06, 0.10, and 0.01. The *F*_st_ ranges among *L. chinense* populations were 0.042–0.94, 0–0.57, and 0–0.21, while within *L. tulipifera* populations, they ranged from 0–0.60, 0.08–0.45, and 0.09–0.6.

### 3.5 Ancestral Populations and Divergence Time Analysis in *Liriodendron*

The DIYABC analysis confirmed that *LtDHN2* primarily supported scenario D and secondarily supported scenario C, with posterior probabilities of 0.4726 and 0.4893, respectively (Table 3). This finding suggests that *Liriodendron* originated in North America during the late Tertiary period (12.6–12.3 Ma), followed by the divergence of *L. chinense* populations in western and eastern China (2.19–2.23 Ma). *LtDHN3* strongly favored scenario F, with a posterior probability of 0.7740, indicating that an eastern ancestral population gave rise to *L. tulipifera* around 13.3 (13.1–13.) Ma and western *L. chinense* around 2.19 Ma. Meanwhile, *LtTLP11* predominantly supported scenario J (0.4978), proposing a western ancestral origin, with *L. tulipifera* evolving at approximately 11.4 Ma and eastern *L. chinense* diverging at 2.19 Ma. Additionally, *LtDHN2* lent some support to scenarios F and J, while *LtDHN3* supported scenario J and *LtTLP11* also provided support for scenarios C, D, and F.

**TABLE 3:** Summary of posterior probability of 12 putative scenarios of *Liriodendron* using DIYABC analysis.

Scenarios ID	*LtDHN2*	*LtDHN3*	*LtTLP11*
A	0.0000 [0.0000, 0.0000]	0.0000 [0.0000, 0.0000]	0.0000 [0.0000, 0.0000]
B	0.0000 [0.0000, 0.0000]	0.0000 [0.0000, 0.0000]	0.0000 [0.0000, 0.0000]
C	0.4726 [0.3653, 0.5799]	0.0000 [0.0000, 1.0000]	0.1727 [0.0000, 0.4141]
D	0.4893 [0.3830, 0.5957]	0.0000 [0.0000, 1.0000]	0.0741 [0.0000, 0.3110]
E	0.0000 [0.0000, 0.0000]	0.0000 [0.0000, 0.0000]	0.0000 [0.0000, 0.0000]
F	0.0135 [0.0000, 0.1141]	0.7740 [0.0000, 1.0000]	0.2554 [0.0000, 0.5800]
G	0.0000 [0.0000, 0.0000]	0.0000 [0.0000, 0.0000]	0.0000 [0.0000, 0.0000]
H	0.0000 [0.0000, 0.0000]	0.0000 [0.0000, 0.0000]	0.0000 [0.0000, 0.0000]
I	0.0000 [0.0000, 0.0000]	0.0000 [0.0000, 0.0000]	0.0000 [0.0000, 0.0000]
J	0.0246 [0.0000, 0.1253]	0.2260 [0.0000, 1.0000]	0.4978 [0.2677, 0.7278]
K	0.0000 [0.0000, 0.0000]	0.0000 [0.0000, 0.0000]	0.0000 [0.0000, 0.0000]
L	0.0000 [0.0000, 0.0000]	0.0000 [0.0000, 0.0000]	0.0000 [0.0000, 0.0000]

### 3.6 The Historical Gene Flow of Three Stress‐Related Single‐Copy Genes in *Liriodendron*

**FIGURE 4:**
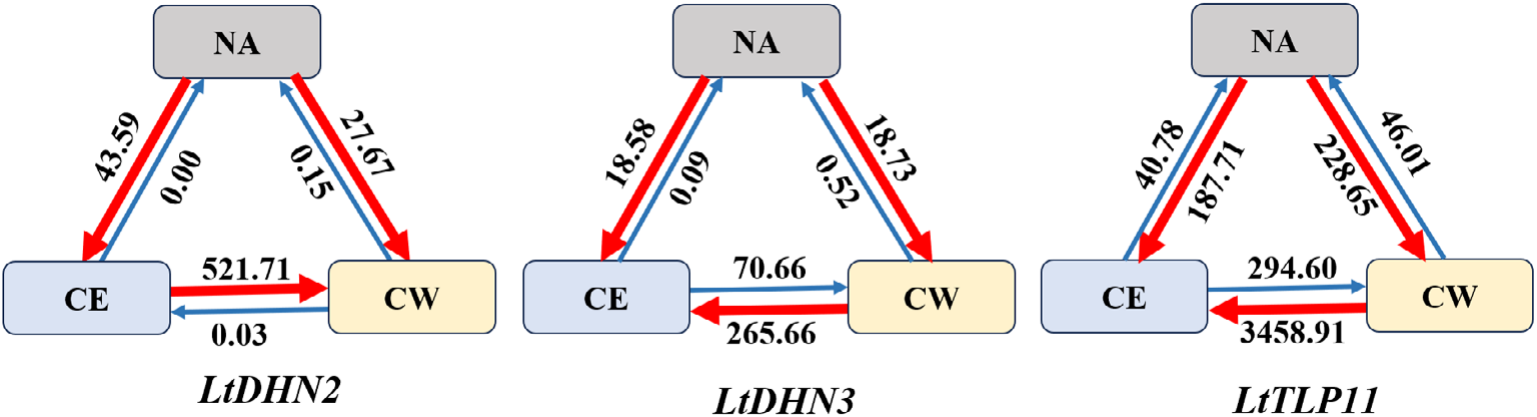
Direction and level of gene flow between pairs of major population regions of *Liriodendron*. CE, *L. chinense* populations from Eastern China; CW, *L. chinense* populations from Western China; NA, *L. tulipifera* populations from North America. Arrows and numbers on arrows represent the direction and level of gene flow, respectively.

The results of the historical gene flow analysis are shown in Figure 4. At the *LtDHN2* and *LtDHN3* loci, the NA population exhibited higher historical gene flow towards the CE and CW Chinese populations than in the opposite direction. Conversely, for the remaining gene, *LtTLP11*, the pattern was reversed, with higher historical gene flow from the CE and CW populations to the NA. For *LtDHN2*, lower historical gene flow from the CW to the CE population was noted, a trend not mirrored in the opposite direction. Conversely, for *LtDHN3* and *LtTLP11*, the historical gene flow was comparatively higher in the CW‐to‐CE trajectory. These results indicated that *L. chinense* populations from Eastern China, *L. chinense* populations from Western China, and *L. tulipifera* populations have all acted as source populations for each other.

### 3.7 Population History Analysis in *Liriodendron*

**FIGURE 5:**
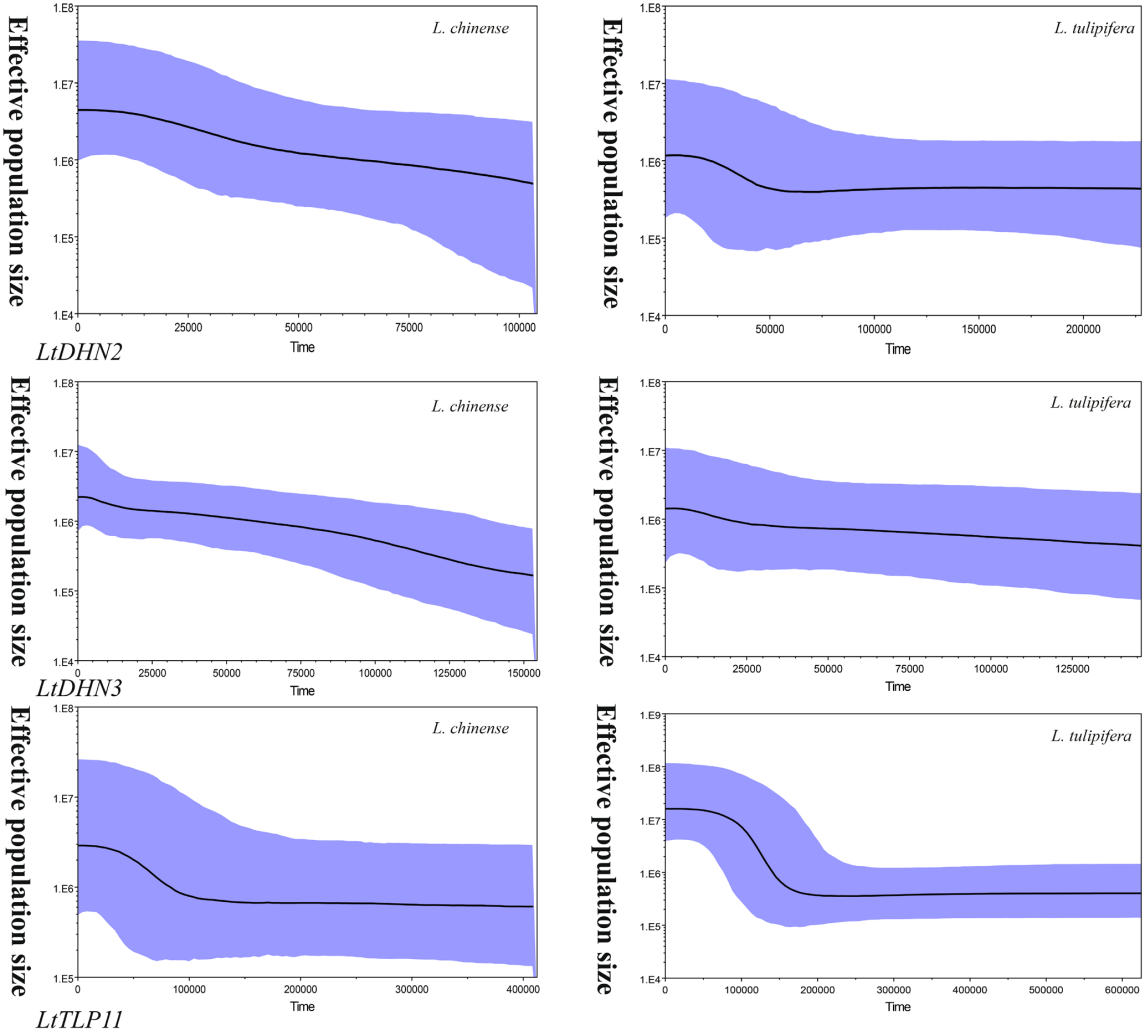
Bayesian Skyline plots showing changes in effective population size from the *LtDHN2*, *LtDHN3*, and *LtTLP11 sequences* of *Liriodendron chinense* and *Liriodendron tulipifera* .

The ranges of evolutionary rates of *LtDHN2*, *LtDHN3*, and *LtTLP11* were 8.5 × 10^−9^ to 1.3 × 10^−8^, 1.3 × 10^−8^ to 1.9 × 10^−8^, and 8.0 × 10^−9^ to 1.2 × 10^−8^, respectively. Figure 5 illustrates the demographic history inferred from the *LtDHN2*, *LtDHN3*, and *LtTLP11* gene sequences. For *L. chinense* , the expression of *LtDHN2* and *LtDHN3* suggested very slow growth. The *LtTLP11* gene indicated rapid effective population size growth between 0.05 and 0.13 Ma, with stability in other periods. For *L. tulipifera* , the *LtDHN2* gene sequences showed a rapid increase between 0.025 and 0.06 Ma, followed by a plateau. The *LtDHN3* gene expression suggested a very slow growth. The *LtTLP11* gene showed rapid growth between 0.07 and 0.15 Ma, with stability during other periods.

## 4 Discussion

### 4.1 Functional Enrichment Analysis of Single‐Copy Genes in *Liriodendron*

Comprehensive genomic analyses of *Liriodendron* revealed conserved adaptive mechanisms through 8667 single‐copy orthologs, with the most enriched functions in energy metabolism, photosynthesis, and stress responses. Both adenyl ribonucleotide binding and adenyl nucleotide binding (MF) represent conserved molecular recognition mechanisms for adenosine‐phosphate moieties (Dan et al. 2010). The thylakoid lumen and plastid thylakoid lumen (CC) are specialized photosynthetic compartments that optimize light‐use efficiency under fluctuating climatic conditions (Inagaki 2022; Liu and Last 2017). Response to herbicide triggers defensive adaptations against phytotoxic chemicals, while benzene‐containing compound metabolism processes aromatic molecules through specialized biochemical transformations (Salas‐Perez et al. 2018; Wang et al. 2024). The three KEGG classifications, A09120 genetic information processing, B09182 protein families: genetic information processing, and B09122 translation, represent fundamental cellular systems for maintaining and executing genetic instructions (Huang et al. 2022).

Purifying selection on energy metabolism (adenyl ribonucleotide binding and adenyl nucleotide binding) and photosynthesis (thylakoid lumen and plastid thylakoid lumen) has maintained the long‐term stability of core genes, enabling deep‐time genetic stability and adaptive fine‐tuning for fundamental survival capacity (Gollan et al. 2021; Hewitt 2004). These conserved adaptations enabled *Liriodendron* to endure Pleistocene glaciations (0.0117–2.58 Ma), where habitat bottlenecks intensified selective pressures (Hofreiter and Stewart 2009). Earlier, during the Miocene cooling (5.3–23 Ma), montane refugia drove adaptations to reduced light intensity, further refining photosynthetic efficiency (Adie and Lawes 2023; Järvi et al. 2013; Wen et al. 2023). The evolution of herbicide response and benzene‐containing compound metabolic processes in *Liriodendron* has formed detoxification. During the Pleistocene (0.01–2.6 Ma), glacial cycles and soil toxicity prompted ribosomal proteins (translation) and DNA repair genes to mitigate oxidative damage from cold/UV stress (Yamori et al. 2014). During the Oligocene and Miocene, global cooling coupled with habitat fragmentation imposed purifying selection on translation machinery to maintain protein synthesis fidelity under energy constraints (Pimentel et al. 2017). In *Liriodendron*, the conservation of these information‐processing pathways underscores their critical role in stress adaptation.

### 4.2 Genetic Diversity and Differentiation of Three Stress‐Related Single‐Copy Genes in *Liriodendron*

Genetic diversity analysis identified a higher SNP density ranging from 1/15.80 to 1/19.45 bp across three stress‐related single‐copy genes in *Liriodendron*. This SNP density exceeded 1/100 bp in *Quercus robur* L., 1/68 bp in cultivated and wild grapevines in Croatia, and 1/53 bp in Corylus (Marinov et al. 2024; Plomion et al. 2018; Yang et al. 2023). The nucleotide diversity metrics (*π* = 0.0114–0.0187, *θ* = 0.0116–0.0148) exhibited higher diversity than *Picea abies* (*π* = 0.0079) but lower diversity than *Rhododendron meddianum* (*π* = 0.0772), aligned slightly lower than the genome‐wide mean of 0.0156 per site in *L. chinense* (Chen et al. 2018; Zhang et al. 2021). Collectively, these three stress‐related genes represent genetically highly variable regions within *Liriodendron*. This elevated variation likely provides a genetic foundation for coping with environmental stress, serving as an important manifestation of the genus' adaptability and evolutionary potential.

The exceptionally high proportion of genetic variation between *L. chinense* and *L. tulipifera* underscored strong interspecific divergence driven by local adaptation and restricted gene flow following speciation. Minimal population structure within *Liriodendron* (0.85%–5.42% variation) and low intraspecific differentiation (*F*_st_ of *L. chinense* East–West: 0.01–0.10) reflected recent glacial expansion and recurrent gene flow across geographical barriers (Dayton et al. 2017). *LtDHN2* exhibited extreme population differentiation in *L. chinense* , with a higher *F*_st_ value of 0.94 between the YJ and WYS populations, implying altitudinal adaptation within western refugia (Khan et al. 2021).

### 4.3 Divergent SNPs Driving Species‐Specific Molecular Adaptations in *Liriodendron*

The stress‐related genes in *Liriodendron* exhibited profound genetic divergence between *L. chinense* and *L. tulipifera* , with completely different SNPs underlying the strong local adaptation (Table 4). For *LtDHN2*, the substitution of histidine (H) in *L. chinense* with glutamine (Q) in *L. tulipifera* at position 21 removed a critical metal‐binding residue, likely reducing ROS scavenging, as histidine in dehydrins binds Cu^2+^/Zn^2+^ to maintain redox balance under drought or cold stress (Yu 2025). Similarly, replacing glutamic acid (E) in *L. chinense* with aspartic acid (D) in *L. tulipifera* at position 99 shortened the carboxyl side chain in *L. tulipifera* , potentially lowering the affinity for divalent cations such as Ca^2+^, which helped regulate chaperone activity and membrane stability (Close 1996). Swapping the hydrophilic aspartate (D) in *L. chinense* for hydrophobic valine (V) at position 31 may alter protein folding or interactions in *L. tulipifera* (Pantelić et al. 2022). Changing proline (P) in *L. chinense* to threonine (T) at position 123 disrupted a proline‐rich turn, likely reducing protein flexibility in *L. tulipifera* (Smith and Graether 2022). Replacing asparagine (N) in *L. chinense* with aspartic acid (D) at position 132 added a negative charge, possibly strengthening interactions with positively charged molecules in *L. tulipifera* (Nelson et al. 2021). Replacing alanine (A) in *L. chinense* with valine (V) in *L. tulipifera* at position 135 boosted hydrophobicity, possibly strengthening membrane binding during dehydration stress (Koag et al. 2009). Inserting ‘TV’ (Threonine, Valine) at positions 137 and 138 in *L. tulipifera* may create a new kinase target (Alsheikh et al. 2003).

**TABLE 4:** Divergence of nucleotide and corresponding amino acid in genes *LtDHN2* and *LtDHN3* between *Liriodendron chinense* and *Liriodendron tulipifera* .

Gene	Nucleotide position	Nucleotide variation	*L. chinense* codon	*L. tulipifera* codon	Codon position	Amino acid change
*LtDHN2*	63	T → A	CAT	CAA	21	His (H) → Gln (Q)
	92	A → T	GAC	GTC	31	Asp (D) → Val (V)
	1196	G → T	GAG	GAT	99	Glu (E) → Asp (D)
	1266	C → A	CCC	ACC	123	Pro (P) → Thr (T)
	1296	A → G	AAC	GAC	132	Asn (N) → Asp (D)
	1306	C → T	GCG	GTG	135	Ala (A) → Val (V)
	1308	– → A	—	ACT	137	– → Thr (T)
	1309	– → C				
	1310	– → T				
	1311	– → G	—	GTG	138	– → Ala (A)
	1312	– → T				
	1313	– → G				
*LtDHN3*	79	– → G	—	GCC	9	– → Thr (T)
	80	– → C				
	81	– → C				
	3184	– → G	—	GAA	74	– → Glu (E)
	3185	– → A				
	3186	– → A				
	3381	G → C	ATG	ATC	139	Met (M) → ATC (I)
	3406	C → T	CCA	TCA	148	Pro (P) → Ser (S)
	3447	T → A	GAT	GAA	161	Asp (D) → Glu (E)
	3565	A → —	AAG	—	201	Lys (K) → —
	3566	A → —				
	3567	G → —				
	3568	T → C	TAC	CAC	202	Tyr (Y) → His (H)

*Note:* Nucleotide position is based on the nucleotide sequence of the *L. chinense* and *L. tulipifera* ; A dash (–) indicates the absence of a nucleotide and amino acid at that position.

For *LtDHN3*, the “A” inserted at position 9 in *L. tulipifera* created stress signaling phosphorylation sites, while the expanded polyglutamate tract (additional “E”) at position 74 enhanced water retention (Graether and Boddington 2014). The substitution of methionine (M) in *L. chinense* with isoleucine (I) in *L. tulipifera* at position 139 of the K‐segment may optimize membrane binding and enhance drought adaptation (Murray and Graether 2022). The substitution of proline (P) in *L. chinense* with serine (S) in *L. tulipifera* at position 148 altered the phosphorylation dynamics (Hanin et al. 2011). A shift from aspartic acid (D) in *L. chinense* to glutamic acid (E) in *L. tulipifera* at position 161 enhanced ion binding (Decena et al. 2021). The replacement of C‐terminal tyrosine (K, Y) in *L. chinense* with histidine (–, H) in *L. tulipifera* at positions 201 and 202 conferred metal‐coordinating capacity (Zhang et al. 2024). *L. chinense* retained core functions in oxidative stress protection, such as metal and cation binding, adapted to high‐altitude mountainous habitats. *L. tulipifera* shifted towards enhanced membrane stability through increased hydrophobicity and optimized membrane binding, alongside innovative signaling pathways via novel phosphorylation sites, to cope with episodic drought stress.

### 4.4 The Origin and Evolutionary History of *Liriodendron*

The DIYABC analyses, combined with fossil evidence, supported four plausible evolutionary scenarios (C, D, F, J). These scenarios, along with historical gene flow patterns, suggested that both *L. tulipifera* and the eastern and western populations of *L. chinense* served as ancestral populations. The results from these scenarios indicated that the divergence between *L. chinense* and *L. tulipifera* occurred approximately 11.4–13.4 Ma, consistent with other studies placing their divergence during the Miocene (10–16 Ma) (Cheng and Li 2018; Shen, Tu, et al. 2022; Shen, Xia, et al. 2022). Historical gene flow among the three gene pools suggested multiple asymmetric migrations between the two continents. During the warm Cenomanian–Turonian period (80–100 Ma), there was a significant surge in biodiversity, accompanied by the divergence of *Liriodendron* from other Magnoliids (Pucéat et al. 2003). The existence of the Bering Land Bridge and the intermittent connectivity of the North Atlantic Land Bridge facilitated the dispersal of plants within *Liriodendron* (Zhou et al. 2020). Despite climatic fluctuations during the Late Cretaceous and Early Paleogene, gene flow persisted between continents. However, by the Early Eocene, the breakup of the North Atlantic Land Bridge halted *Liriodendron* exchange between North America, Europe, and Asia (Muellner‐Riehl and Rojas‐Andrés 2022). Later, during the Late Miocene, global cooling forced temperate plants, including *Liriodendron*, to retreat southward and prevented further migration via the Bering Land Bridge (Graham 2018).

With the cooling of the Late Tertiary and the mass extinctions of the Quaternary Ice Age, *Liriodendron* species in other regions gradually became extinct, leaving only *L. chinense* and *L. tulipifera* (Yang et al. 2019). The divergence between the eastern and western populations of *L. chinense* was estimated to have occurred between 2.19 and 2.23 Ma. Although the exact onset of the earliest glacial period in Quaternary China remains uncertain, this divergence time frame aligns with the major climatic shifts (Jin et al. 2020). The Quaternary fossil record demonstrated a dramatic decline in the genus, with *L. tulipifera* fossils found only along the Atlantic coast of the eastern United States (Yang et al. 2016). Populations experienced cyclical declines during glacial phases but expanded during interstadials in response to ice age transitions and Pleistocene glacial–interglacial cycles.

In *L. chinense* , rapid population expansion inferred from *LtTLP11* (0.05–0.13 Ma) coincided with the Early Last Glacial (MIS 4; 0.057–0.071 Ma), the Last Interglacial (MIS 5a–5d; 0.071–0.116 Ma), and the Last Interglacial Thermal Maximum (MIS 5e; 0.116–0.130 Ma). In contrast, the slow growth patterns of *LtDHN2* and *LtDHN3* indicated prolonged glacial‐era bottlenecks under cold, arid conditions (Hernández‐Langford et al. 2020; Landais et al. 2016). Similarly, in *L. tulipifera* , rapid expansions detected in *LtDHN2* (0.025–0.06 Ma) and *LtTLP11* (0.07–0.15 Ma) corresponded to distinct warmer intervals. Specifically, the expansion in *LtDHN2* aligned with the MIS 3 interstadial resilience phase (0.025–0.057 Ma), while the expansion in *LtTLP11* matched the fluctuating substages of the Last Interglacial (MIS 5a–5d; 0.071–0.116 Ma and MIS 5e; 0.116–0.130 Ma), and the late Penultimate Glacial Maximum warming phase (MIS 6; 0.130–0.150 Ma). Conversely, the slow growth of *LtDHN3* suggested persistent environmental constraints during glacial periods (McLachlan et al. 2005).

## 5 Conclusion

Comprehensive genomic analyses of *Liriodendron* revealed conserved adaptive mechanisms through 8667 single‐copy orthologs enriched in energy metabolism, photosynthesis, and stress response. Notably, the stress‐related genes *LtDHN2*, *LtDHN3*, and *LtTLP11* in *Liriodendron* exhibited exceptionally high SNP density (1/15.80–1/19.45 bp) and significant interspecific differentiation. The evolutionary histories reconstructed by the three stress‐related single‐copy genes revealed that both *L. tulipifera* and the eastern and western populations of *L. chinense* were ancestral populations. The divergence time between *L. chinense* and *L. tulipifera* occurred approximately 11.4–13.4 Ma. The split between the eastern and western populations of *L. chinense* occurred 2.19–2.23 Ma. Demographic reconstructions revealed that *L. chinense* exhibited expansion during the Early Last Glacial and the Last Interglacial, while *L. tulipifera* demonstrated a resilience phase during the warmer MIS 3 interstadial resilience phase, the fluctuating substages of the Last Interglacial, and the late Penultimate Glacial Maximum warming phase. Consequently, *L. chinense* retained core functions in oxidative stress protection to adapt to high‐altitude mountainous habitats; *L. tulipifera* enhanced membrane stability to cope with episodic drought stress. While this study provided how paleogeographic constraints, climatic fluctuations, and molecular adaptation shaped *Liriodendron*'s disjunct distribution and genetic architecture, it also acknowledged two key limitations of restricted population sampling across its range and reliance solely on three adaptive single‐copy genes. Future research should integrate whole‐genome sequencing with paleoclimate simulations across an expanded set of representative populations. This integrated approach is essential to systematically reconstruct the evolutionary trajectory of *Liriodendron* and to functionally characterize fixed SNPs that distinguish *L. chinense* and *L. tulipifera* , thereby enabling precision breeding of key adaptive traits.

## Author Contributions

**Yanli Cheng:** data curation (equal), investigation (equal), methodology (lead), software (equal), visualization (equal), writing – original draft (equal), writing – review and editing (equal). **Heyang Yuan:** data curation (equal), formal analysis (equal), methodology (equal), software (equal). **Lichun Yang:** software (equal), visualization (equal). **Xi Chen:** formal analysis (equal), software (equal). **Huogen Li:** conceptualization (equal), funding acquisition (lead), investigation (equal), project administration (equal), resources (equal), writing – review and editing (equal).

## Conflicts of Interest

The authors declare no conflicts of interest.

## Supporting information


Appendix A.

Appendix B.

**Table S1:** ece372182‐sup‐0003‐TableS1.docx.

## Data Availability

The identified single‐copy genes in *Liriodendron* and *LtDHN2*, *LtDHN3*, and *LtTLP11* gene sequences of *Liriodendron* populations were submitted to the Dryad Digital Repository at https://doi.org/10.5061/dryad.0vt4b8h8n. Additional supporting information, including Table S1 the primers for *LtDHN2*, *LtDHN3*, and *LtTLP11* cloning; Appendix A the 5′RACE, 3′RACE, and cDNA sequences of *LtDHN2*, *LtDHN3*, and *LtTLP11* genes; and Appendix B the RNA sequence and amino acid translation of these genes, can be found online in the Supporting Information section.

## References

[ece372182-bib-0001] Adie, H. , and M. J. Lawes . 2023. “Solutions to Fire and Shade: Resprouting, Growing Tall and the Origin of Eurasian Temperate Broadleaved Forest.” Biological Reviews 98, no. 2: 643–661. 10.1111/brv.12923. 36444419

[ece372182-bib-0002] Alsheikh, M. K. , B. J. Heyen , and S. K. Randall . 2003. “Ion Binding Properties of the Dehydrin ERD14 Are Dependent Upon Phosphorylation.” Journal of Biological Chemistry 278, no. 42: 40882–40889. 10.1074/jbc.M307151200. 12917402

[ece372182-bib-0003] Bell, W. A. 1957. “Flora of the Upper Cretaceous Nanaimo Group of Vancouver Island, British Columbia.” Geological Survey of Canada Memoir 293: 1–84. 10.4095/101457.

[ece372182-bib-0004] Benton, M. J. , P. Wilf , and H. Sauquet . 2022. “The Angiosperm Terrestrial Revolution and the Origins of Modern Biodiversity.” New Phytologist 233, no. 5: 2017–2035. 10.1111/nph.17822. 34699613

[ece372182-bib-0005] Cevallos‐Ferriz, S. R. S. , and R. A. Stockey . 1990. “Vegetative Remains of the Magnoliaceae From the Princeton Chert (Middle Eocene) of British Columbia.” Canadian Journal of Botany 68, no. 6: 1327–1339. 10.1139/b90-169.

[ece372182-bib-0006] Chaudhry, S. , and G. P. S. Sidhu . 2022. “Climate Change Regulated Abiotic Stress Mechanisms in Plants: A Comprehensive Review.” Plant Cell Reports 41, no. 1: 1–31. 10.1007/s00299-021-02759-5. 34351488

[ece372182-bib-0007] Chen, C. , Y. Wu , J. Li , et al. 2023. “TBtools‐II: A ‘One for All, All for One’ Bioinformatics Platform for Biological Big‐Data Mining.” Molecular Plant 16, no. 11: 1733–1742. 10.1016/j.molp.2023.09.010. 37740491

[ece372182-bib-0008] Chen, J. , Z. Hao , X. Guang , et al. 2018. “ *Liriodendron* Genome Sheds Light on Angiosperm Phylogeny and Species–Pair Differentiation.” Nature Plants 5, no. 1: 18–25. 10.1038/s41477-018-0323-6. 30559417 PMC6784881

[ece372182-bib-0010] Cheng, Y. , and H. Li . 2018. “Interspecies Evolutionary Divergence in *Liriodendron*, Evidence From the Nucleotide Variations of *LcDHN‐Like* Gene.” BMC Evolutionary Biology 18, no. 1: 195. 10.1186/s12862-018-1318-7. 30567488 PMC6300021

[ece372182-bib-0011] Close, T. J. 1996. “Dehydrins: Emergence of a Biochemical Role of a Family of Plant Dehydration Proteins.” Physiologia Plantarum 97, no. 4: 795–803. 10.1111/j.1399-3054.1996.tb00546.x.

[ece372182-bib-0012] Cornuet, J.‐M. , F. Santos , M. A. Beaumont , et al. 2008. “Inferring Population History With DIY ABC: A User‐Friendly Approach to Approximate Bayesian Computation.” Bioinformatics 24, no. 23: 2713–2719. 10.1093/bioinformatics/btn514. 18842597 PMC2639274

[ece372182-bib-0013] Dan, A. , Y. Ofran , and Y. Kliger . 2010. “Large‐Scale Analysis of Secondary Structure Changes in Proteins Suggests a Role for Disorder‐to‐Order Transitions in Nucleotide Binding Proteins.” Proteins: Structure, Function, and Bioinformatics 78, no. 2: 236–248. 10.1002/prot.22531. 19676113

[ece372182-bib-0014] Dayton, J. , M. Ledwoń , J.‐M. Paillisson , N. Atamas , and P. Szczys . 2017. “Genetic Diversity and Population Structure of the Eurasian Whiskered Tern ( *Chlidonias hybrida hybrida* ), a Species Exhibiting Range Expansion.” Waterbirds 40, no. 2: 105–117. 10.1675/063.040.0203.

[ece372182-bib-0015] De Vaz Sousa, D. , M. Greve , and K. C. Oberlander . 2024. “Friends Without Benefits: Extensive Cytotype Sympatry and Polyploid Persistence in an African Geophyte.” American Journal of Botany 111, no. 8: e16291. 10.1002/ajb2.16291. 38439133

[ece372182-bib-0016] Decena, M. A. , S. Gálvez‐Rojas , F. Agostini , et al. 2021. “Comparative Genomics, Evolution, and Drought‐Induced Expression of Dehydrin Genes in Model Brachypodium Grasses.” Plants 10, no. 12: 2664. 10.3390/plants10122664. 34961135 PMC8709310

[ece372182-bib-0017] Ding, W. , D. Silvestro , R. E. Onstein , M. Wu , Z. Zhou , and Y. Xing . 2025. “The Stepwise Rise of Angiosperm‐Dominated Terrestrial Ecosystems.” Biological Reviews 100: 2131–2149. 10.1111/brv.70039. 40443389 PMC12407066

[ece372182-bib-0018] Dong, S. , Y. Xiao , H. Kong , et al. 2019. “Nuclear Loci Developed From Multiple Transcriptomes Yield High Resolution in Phylogeny of Scaly Tree Ferns (Cyatheaceae) From China and Vietnam.” Molecular Phylogenetics and Evolution 139: 106567. 10.1016/j.ympev.2019.106567. 31330266

[ece372182-bib-0019] Excoffier, L. , and H. E. L. Lischer . 2010. “Arlequin Suite ver 3.5: A New Series of Programs to Perform Population Genetics Analyses Under Linux and Windows.” Molecular Ecology Resources 10, no. 3: 564–567. 10.1111/j.1755-0998.2010.02847.x. 21565059

[ece372182-bib-0020] Feng, L. , S. Wei , and Y. Li . 2024. “Thaumatin‐Like Proteins in Legumes: Functions and Potential Applications—A Review.” Plants 13, no. 8: 1124. 10.3390/plants13081124. 38674533 PMC11055134

[ece372182-bib-0021] Fetter, K. C. 2014. “Migration, Adaptation, and Speciation—A Post‐Glacial History of the Population Structure, Phylogeography, and Biodiversity of *Liriodendron tulipifera* L. (Magnoliaceae).” Master's Thesis, University of North Carolina.

[ece372182-bib-0022] Friis, E. M. , P. R. Crane , and K. R. Pedersen . 2025. “The Cretaceous Diversification of Angiosperms: Perspectives From Mesofossils.” Geological Society, London, Special Publications 544, no. 1: 393–432. 10.1144/SP544-2023-170.

[ece372182-bib-0023] Frumin, S. I. , and E. M. Friis . 1996. “Liriodendroid Seeds From the Late Cretaceous of Kazakhstan and North Carolina, USA.” Review of Palaeobotany and Palynology 94, no. 1–2: 39–55. 10.1016/0034-6667(95)00136-0.

[ece372182-bib-0024] Geissert, F. , H. J. Gregor , and D. H. Mai . 1990. Die ‘Saubaggerflora’, eine Frucht‐ und Samenflora aus dem Grenzbereich Miozän‐Pliozän von Sessenheim im Elsaß (Frankreich). Kanzler.

[ece372182-bib-0025] Gollan, P. J. , A. Trotta , and A. A. Bajwa . 2021. “Characterization of the Free and Membrane‐Associated Fractions of the Thylakoid Lumen Proteome in *Arabidopsis thaliana* .” International Journal of Molecular Sciences 22, no. 15: 8126. 10.3390/ijms22158126. 34360890 PMC8346976

[ece372182-bib-0026] Graether, S. P. , and K. F. Boddington . 2014. “Disorder and Function: A Review of the Dehydrin Protein Family.” Frontiers in Plant Science 5: 576. 10.3389/fpls.2014.00576. 25400646 PMC4215689

[ece372182-bib-0027] Graham, A. 2018. “The Role of Land Bridges, Ancient Environments, and Migrations in the Assembly of the North American Flora.” Journal of Systematics and Evolution 56, no. 5: 405–429. 10.1111/jse.12302.

[ece372182-bib-0028] Hanin, M. , F. Brini , C. Ebel , Y. Toda , S. Takeda , and K. Masmoudi . 2011. “Plant Dehydrins and Stress Tolerance: Versatile Proteins for Complex Mechanisms.” Plant Signaling & Behavior 6, no. 10: 1503–1509. 10.4161/psb.6.10.17088. 21897131 PMC3256378

[ece372182-bib-0029] Hernández‐Langford, D. G. , M. E. Siqueiros‐Delgado , and E. Ruíz‐Sánchez . 2020. “Nuclear Phylogeography of the Temperate Tree Species *Chiranthodendron pentadactylon* (Malvaceae): Quaternary Relicts in Mesoamerican Cloud Forests.” BMC Evolutionary Biology 20: 1–14. 10.1186/s12862-020-01605-8. 32306974 PMC7168997

[ece372182-bib-0030] Hewitt, G. M. 2004. “Genetic Consequences of Climatic Oscillations in the Quaternary.” Philosophical Transactions of the Royal Society of London. Series B, Biological Sciences 359, no. 1442: 183–195. 10.1098/rstb.2003.1388. 15101575 PMC1693318

[ece372182-bib-0031] Hofreiter, M. , and J. Stewart . 2009. “Ecological Change, Range Fluctuations and Population Dynamics During the Pleistocene.” Current Biology 19, no. 14: R584–R594. 10.1016/j.cub.2009.06.030. 19640497

[ece372182-bib-0032] Hu, Y. , C. Gong , Z. Yang , et al. 2025. “Functional Divergence of Plant‐Derived *Thaumatin‐Like* Protein Genes in Two Closely Related Whitefly Species.” Advanced Science 12, no. 16: 2502193. 10.1002/advs.202502193. 40019366 PMC12021119

[ece372182-bib-0033] Hu, Y. , J. Wang , L. Liu , et al. 2025. “Evolutionary History of Magnoliid Genomes and Benzylisoquinoline Alkaloid Biosynthesis.” Nature Communications 16, no. 1: 4039. 10.1038/s41467-025-59343-8. PMC12041406 40301376

[ece372182-bib-0034] Huang, J. , B. Tang , R. Ren , et al. 2022. “Understanding the Potential Gene Regulatory Network of Starch Biosynthesis in Tartary Buckwheat by RNA‐Seq.” International Journal of Molecular Sciences 23, no. 24: 15774. 10.3390/ijms232415774. 36555415 PMC9779217

[ece372182-bib-0035] Inagaki, N. 2022. “Processing of D1 Protein: A Mysterious Process Carried out in Thylakoid Lumen.” International Journal of Molecular Sciences 23, no. 5: 2520. 10.3390/ijms23052520. 35269663 PMC8909930

[ece372182-bib-0036] Järvi, S. , P. J. Gollan , and E.‐M. Aro . 2013. “Understanding the Roles of the Thylakoid Lumen in Photosynthesis Regulation.” Frontiers in Plant Science 4: 1–14. 10.3389/fpls.2013.00434. 24198822 PMC3813922

[ece372182-bib-0037] Jin, H. , J. Vandenberghe , D. Luo , et al. 2020. “Quaternary Permafrost in China: Framework and Discussions.” Quaternary 3, no. 4: 32. 10.3390/quat3040032.

[ece372182-bib-0039] Khan, M. M. H. , M. Y. Rafii , S. I. Ramlee , M. Jusoh , M. Al Mamun , and J. Halidu . 2021. “DNA Fingerprinting, Fixation‐Index (*F* _st_), and Admixture Mapping of Selected Bambara Groundnut (*Vigna subterranea* [L.] Verdc.) Accessions Using ISSR Markers System.” Scientific Reports 11, no. 1: 14527. 10.1038/s41598-021-93867-5. 34267249 PMC8282841

[ece372182-bib-0040] Koag, M. C. , S. Wilkens , R. D. Fenton , J. Resnik , E. Vo , and T. J. Close . 2009. “The K‐Segment of Maize DHN1 Mediates Binding to Anionic Phospholipid Vesicles and Concomitant Structural Changes.” Plant Physiology 150, no. 3: 1503–1514. 10.1104/pp.109.136697. 19439573 PMC2705017

[ece372182-bib-0041] Kumar, S. , G. Stecher , M. Suleski , M. Sanderford , S. Sharma , and K. Tamura . 2024. “MEGA12: Molecular Evolutionary Genetic Analysis Version 12 for Adaptive and Green Computing.” Molecular Biology and Evolution 41, no. 12: msae263. 10.1093/molbev/msae263. 39708372 PMC11683415

[ece372182-bib-0042] Landais, A. , V. Masson‐Delmotte , and E. Capron . 2016. “How Warm Was Greenland During the Last Interglacial Period?” Climate of the Past 12, no. 9: 1933–1948. 10.5194/cp-12-1933-2016.

[ece372182-bib-0043] LeBlanc, D. , J. Maxwell , N. Pederson , A. Berland , and T. Mandra . 2020. “Radial Growth Responses of Tulip Poplar ( *Liriodendron tulipifera* ) to Climate in the Eastern United States.” Ecosphere 11, no. 10: e03203. 10.1002/ecs2.3203.

[ece372182-bib-0044] Librado, P. , and J. Rozas . 2009. “DnaSP v5: A Software for Comprehensive Analysis of DNA Polymorphism Data.” Bioinformatics 25, no. 11: 1451–1452. 10.1093/bioinformatics/btp187. 19346325

[ece372182-bib-0045] Liu, J. , and R. L. Last . 2017. “A Chloroplast Thylakoid Lumen Protein Is Required for Proper Photosynthetic Acclimation of Plants Under Fluctuating Light Environments.” Proceedings of the National Academy of Sciences of the United States of America 114, no. 38: E8110–E8117. 10.1073/pnas.1712206114. 28874535 PMC5617312

[ece372182-bib-0046] Marinov, L. , G. Magris , G. Di Gaspero , et al. 2024. “Single Nucleotide Polymorphism (SNP) Analysis Reveals Ancestry and Genetic Diversity of Cultivated and Wild Grapevines in Croatia.” BMC Plant Biology 24, no. 1: 975. 10.1186/s12870-024-05675-4. 39420269 PMC11483961

[ece372182-bib-0047] Maryland Geological Survey , R. S. Bassler , E. W. Berry , et al. 1916. Upper Cretaceous. Johns Hopkins press. 10.5962/bhl.title.22326.

[ece372182-bib-0048] McLachlan, J. S. , J. S. Clark , and P. S. Manos . 2005. “Molecular Indicators of Tree Migration Capacity Under Rapid Climate Change.” Ecology 86, no. 8: 2088–2098. 10.1890/04-1036.

[ece372182-bib-0049] Muellner‐Riehl, A. N. , and B. M. Rojas‐Andrés . 2022. “Biogeography of Neotropical Meliaceae: Geological Connections, Fossil and Molecular Evidence Revisited.” Brazilian Journal of Botany 45, no. 1: 527–543. 10.1007/s40415-021-00770-4.

[ece372182-bib-0050] Murray, M. R. , and S. P. Graether . 2022. “Physiological, Structural, and Functional Insights Into the Cryoprotection of Membranes by the Dehydrins.” Frontiers in Plant Science 13: 886525. 10.3389/fpls.2022.886525. 35574140 PMC9096783

[ece372182-bib-0051] Nakandala, U. , A. Furtado , M. W. Smith , D. C. Williams , and R. J. Henry . 2023. “Phylogenetic Relationships Among Australian Native Citrus Species Based Upon Complete Chloroplast Genomes and Single Copy Nuclear Genes.” Tropical Plants 2, no. 1: 1–9. 10.48130/TP-2023-0021.

[ece372182-bib-0052] Naranjo, J. G. , C. B. Sither , and G. C. Conant . 2024. “Shared Single Copy Genes Are Generally Reliable for Inferring Phylogenetic Relationships Among Polyploid Taxa.” Molecular Phylogenetics and Evolution 196: 108087. 10.1016/j.ympev.2024.108087. 38677353

[ece372182-bib-0053] Nekola, J. C. , M. Nováková , M. Horsák , and C. M. Adema . 2023. “ELAV Intron 8: A Single‐Copy Sequence Marker for Shallow to Deep Phylogeny in *Eupulmonata* Hasprunar & Huber, 1990 and *Hygrophila* Férussac, 1822 (Gastropoda: Mollusca).” Organisms Diversity & Evolution 23, no. 3: 621–629. 10.1007/s13127-022-00587-3.

[ece372182-bib-0054] Nelson, D. L. , M. M. Cox , A. A. Hoskins , et al. 2021. Lehninger Principles of Biochemistry. 8th ed. Macmillan International Higher Education.

[ece372182-bib-0055] Nezu, I. , F. Ishiguri , J. Ohshima , and S. Yokota . 2022. “Relationship Between the Xylem Maturation Process Based on Radial Variations in Wood Properties and Radial Growth Increments of Stems in a Fast‐Growing Tree Species, *Liriodendron tulipifera* .” Journal of Wood Science 68, no. 1: 48. 10.1186/s10086-022-02057-y.

[ece372182-bib-0056] Nie, Z.‐L. , J. Wen , H. Azuma , et al. 2008. “Phylogenetic and Biogeographic Complexity of Magnoliaceae in the Northern Hemisphere Inferred From Three Nuclear Data Sets.” Molecular Phylogenetics and Evolution 48, no. 3: 1027–1040. 10.1016/j.ympev.2008.06.004. 18619549

[ece372182-bib-0057] Pantelić, A. , S. Stevanović , S. M. Komić , N. Kilibarda , and M. Vidović . 2022. “In Silico Characterisation of the Late Embryogenesis Abundant (LEA) Protein Families and Their Role in Desiccation Tolerance in *Ramonda serbica* Panc.” International Journal of Molecular Sciences 23, no. 7: 3547. 10.3390/ijms23073547. 35408906 PMC8998581

[ece372182-bib-0058] Parks, C. R. , N. G. Miller , J. F. Wendel , and K. M. McDougal . 1983. “Genetic Divergence Within the Genus *Liriodendron* (Magnoliaceae).” Annals of the Missouri Botanical Garden 70, no. 4: 658. 10.2307/2398983.

[ece372182-bib-0059] Parks, C. R. , and J. F. Wendel . 1990. “Molecular Divergence Between Asian and North American Species of *Liriodendron* (Magnoliaceae) With Implications for Interpretation of Fossil Floras.” American Journal of Botany 77, no. 10: 1243–1256. 10.1002/j.1537-2197.1990.tb11376.x.

[ece372182-bib-0060] Phan, K. L. 2015. *Liriodendron chinense* . IUCN Red List of Threatened Species. 10.2305/IUCN.UK.2015-2.RLTS.T31284A2803363.en.

[ece372182-bib-0061] Pimentel, M. , M. Escudero , E. Sahuquillo , M. Á. Minaya , and P. Catalán . 2017. “Are Diversification Rates and Chromosome Evolution in the Temperate Grasses (Pooideae) Associated With Major Environmental Changes in the Oligocene‐Miocene?” PeerJ 5: e3815. 10.7717/peerj.3815. 28951814 PMC5611942

[ece372182-bib-0062] Plomion, C. , J.‐M. Aury , J. Amselem , et al. 2018. “Oak Genome Reveals Facets of Long Lifespan.” Nature Plants 4, no. 7: 440–452. 10.1038/s41477-018-0172-3. 29915331 PMC6086335

[ece372182-bib-0063] Pucéat, E. , C. Lécuyer , and S. M. F. Sheppard . 2003. “Thermal Evolution of Cretaceous Tethyan Marine Waters Inferred From Oxygen Isotope Composition of Fish Tooth Enamels.” Paleoceanography 18, no. 2: 1029–1041.

[ece372182-bib-0064] Quassinti, L. , F. Maggi , F. Ortolani , et al. 2019. “Exploring New Applications of Tulip Tree ( *Liriodendron tulipifera* L.): Leaf Essential Oil as Apoptotic Agent for Human Glioblastoma.” Environmental Science and Pollution Research 26, no. 29: 30485–30497. 10.1007/s11356-019-06217-4. 31444719

[ece372182-bib-0065] Rambaut, A. , A. J. Drummond , D. Xie , G. Baele , and M. A. Suchard . 2018. “Posterior Summarization in Bayesian Phylogenetics Using Tracer 1.7.” Systematic Biology 67, no. 5: 901–904. 10.1093/sysbio/syy032. 29718447 PMC6101584

[ece372182-bib-0066] Retallack, G. J. , and D. L. Dilcher . 1986. “Cretaceous Angiosperm Invasion of North America.” Cretaceous Research 7, no. 3: 227–252. 10.1016/0195-6671(86)90027-3.

[ece372182-bib-0067] Salas‐Perez, R. A. , C. A. Saski , R. E. Noorai , et al. 2018. “RNA‐Seq Transcriptome Analysis of *Amaranthus palmeri* With Differential Tolerance to Glufosinate Herbicide.” PLoS One 13, no. 4: e0195488. 10.1371/journal.pone.0195488. 29672568 PMC5908165

[ece372182-bib-0068] Shen, Y. , Y. Cheng , K. Li , and H. Li . 2019. “Integrating Phylogeographic Analysis and Geospatial Methods to Infer Historical Dispersal Routes and Glacial Refugia of *Liriodendron chinense* .” Forests 10, no. 7: 565. 10.3390/f10070565.

[ece372182-bib-0069] Shen, Y. , Z. Tu , Y. Zhang , et al. 2022. “Predicting the Impact of Climate Change on the Distribution of Two Relict *Liriodendron* Species by Coupling the MaxEnt Model and Actual Physiological Indicators in Relation to Stress Tolerance.” Journal of Environmental Management 322: 116024. 10.1016/j.jenvman.2022.116024. 36055092

[ece372182-bib-0070] Shen, Y. , H. Xia , Z. Tu , Y. Zong , L. Yang , and H. Li . 2022. “Genetic Divergence and Local Adaptation of *Liriodendron* Driven by Heterogeneous Environments.” Molecular Ecology 31, no. 3: 916–933. 10.1111/mec.16271. 34773328

[ece372182-bib-0071] Smith, M. A. , and S. P. Graether . 2022. “The Disordered Dehydrin and Its Role in Plant Protection: A Biochemical Perspective.” Biomolecules 12, no. 2: 294. 10.3390/biom12020294. 35204794 PMC8961592

[ece372182-bib-0072] Sun, X. , Y. Ding , M. C. Orr , and F. Zhang . 2020. “Streamlining Universal Single‐Copy Orthologue and Ultraconserved Element Design: A Case Study in Collembola.” Molecular Ecology Resources 20, no. 3: 706–717. 10.1111/1755-0998.13146. 32065730

[ece372182-bib-0073] Szlachtowska, Z. , and M. Rurek . 2023. “Plant Dehydrins and Dehydrin‐Like Proteins: Characterization and Participation in Abiotic Stress Response.” Frontiers in Plant Science 14: 1213188. 10.3389/fpls.2023.1213188. 37484455 PMC10358736

[ece372182-bib-0074] Taylor, D. W. 1990. “Paleobiogeographic Relationships of Angiosperms From the Cretaceous and Early Tertiary of the North American Area.” Botanical Review 56, no. 4: 279–417. 10.1007/BF02995927.

[ece372182-bib-0075] Wang, W. , S. Zhang , T. Gao , and L. Li . 2024. “In‐Situ Treatment of Gaseous Benzene in Fixed‐Bed Biofilter With Polyurethane Foam: Functional Population Response and Benzene Transformation Pathway.” Bioresource Technology 405: 130926. 10.1016/j.biortech.2024.130926. 38824970

[ece372182-bib-0076] Wen, Y. , L. Zhang , A. E. Holbourn , et al. 2023. “CO2 ‐Forced Late Miocene Cooling and Ecosystem Reorganizations in East Asia.” Proceedings of the National Academy of Sciences 120, no. 5: e2214655120. 10.1073/pnas.2214655120. PMC9945954 36689658

[ece372182-bib-0077] Yamori, W. , K. Hikosaka , and D. A. Way . 2014. “Temperature Response of Photosynthesis in C_3_, C_4_, and CAM Plants: Temperature Acclimation and Temperature Adaptation.” Photosynthesis Research 119, no. 1–2: 101–117. 10.1007/s11120-013-9874-6. 23801171

[ece372182-bib-0078] Yang, A. , C. W. Dick , and X. Yao . 2016. “Impacts of Biogeographic History and Marginal Population Genetics on Species Range Limits: A Case Study of *Liriodendron chinense* .” Scientific Reports 6, no. 1: 25632. 10.1038/srep25632. 27162176 PMC4861920

[ece372182-bib-0079] Yang, A. , Y. Zhong , and S. Liu . 2019. “New Insight Into the Phylogeographic Pattern of *Liriodendron chinense* (Magnoliaceae) Revealed by Chloroplast DNA: East–West Lineage Split and Genetic Mixture Within Western Subtropical China.” PeerJ 7: e6355. 10.7717/peerj.6355. 30723627 PMC6361005

[ece372182-bib-0080] Yang, Z. , ed. 2021. Zhongguo Gu Shengwu Zhi: 1951–2018 (Collector's Edition). 1st ed. Science Press.

[ece372182-bib-0081] Yang, Z. , W. Ma , L. Wang , et al. 2023. “Population Genomics Reveals Demographic History and Selection Signatures of Hazelnut (*Corylus*).” Horticulture Research 10, no. 5: uhad065. 10.1093/hr/uhad065. 37249951 PMC10208898

[ece372182-bib-0082] Yu, Z. 2025. “Dehydrins as Key Protector of Plant Abiotic Tolerance: An Update.” Israel Journal of Plant Sciences 1: 1–14. 10.1163/22238980-bja10114.

[ece372182-bib-0083] Zhang, H. , J. Wu , D. Fu , M. Zhang , L. Wang , and M. Gong . 2024. “Prokaryotic Expression, Purification, and the In Vitro and In Vivo Protection Study of Dehydrin WDHN2 From *Triticum aestivum* .” Protoplasma 261, no. 4: 771–781. 10.1007/s00709-024-01933-2. 38342804

[ece372182-bib-0084] Zhang, X. J. , X. F. Liu , D. T. Liu , et al. 2021. “Genetic Diversity and Structure of *Rhododendron meddianum*, a Plant Species With Extremely Small Populations.” Plant Diversity 43, no. 6: 472–479. 10.1016/j.pld.2021.05.005. 35024516 PMC8720705

[ece372182-bib-0085] Zhou, W. , Q. Y. Xiang , and J. Wen . 2020. “Phylogenomics, Biogeography, and Evolution of Morphology and Ecological Niche of the Eastern Asian–Eastern North American *Nyssa* (Nyssaceae).” Journal of Systematics and Evolution 58, no. 5: 571–603. 10.1111/jse.12599.

[ece372182-bib-0086] Zuo, X. , S. Cao , Y. Li , et al. 2023. “Functional Characterization of Dehydrins CpRAB and CpERD and Their Roles in Regulating Cold Resistance of Zucchini Fruit Under High Relative Humidity Storage.” Postharvest Biology and Technology 202: 112387. 10.1016/j.postharvbio.2023.112387.

