# Supplementary material for: Population Genetic Differentiation and Evolutionary History in Liriodendron Revealed by Stress‐Related Single‐Copy Genes
Source: Ecol Evol. 2025 Sep 22;15(9):e72182. doi: 10.1002/ece3.72182 (PMC12453614; doi:10.1002/ece3.72182)
Supplement: Supplementary file 1 — Appendix A. [file ECE3-15-e72182-s001.docx]

**5’ RACE, 3’ RACE and cDNA sequences of *LtDHN2, LtDHN3*and *LtTLP11* genes**

**1 5’ RACE, 3’ RACE and cDNA sequences of *LcDHN2* gene**

**5’ RACE sequence (468 bp)**

GAAAAAAGCAAAAGCAGTGCTCTTCCATCAGTGCTATCATTCATACATTTGTTTCCTTTACTTTTGCTATTTGCGAAGTTTCATTCCGTACATGGCTGAAGAGCAACACCATGATGCGTGCCAGGAGAGTGAGAACGCAGGAGGTGAAATCAAGGACCGTGGGATGTTTGATTTTTTGAGTAAGAAGAAGGAAGAAAAGCCTCAGGAGGAGGCTGTAGTGGCGGAGTTTGAGAAGGTACATGTGTCAGAACCCTATAAGAAGGAGGAGGACGAAAAAGAGGAGAAGCCCATTCACGCTGAGAAGATACAACGGTCAAATAGTAGCAGCTCTAGCTCGTCCAGCGATGAAGAAGAAGGTGAAGGAGGAGAAAAGAAGAAGAAGAAGAAGAAGGGATCATTGAAGGAGAAGTTATTGGGCGAGAAGAAGGAAGAAGAGAAGAAGGTGGCAGAAACGGAGGAGTGTAGAAT

**3’ RACE sequence (345 bp)**

TTGAAGGAGAAGTTATTGGGCGAGAAGAAGGAAGAAGAGAAGAAGGTGGCAGAAACGGAGGAGTGTAGAATTGGAGAAGCCACGGAGGAAAAGAAGGGATTCTTGGAGAAGATCAAAGAGAAGTTGCCTGGGCCCCCGCCCAAGAAGGTGGATGATAGTCACAACAGCACGGCGGCCGCCGCCGAGTGCAGCGCCGAACCACATGGGCACCATGAAGTGGAGGCAGCTAAGGAGAAGAAGGGTCTGTTGGAGAAGATAAAGGAGAAACTGCCTGGCTACCACAAGAATGAAGAGAAGGAGGAGTCTGCCAACCACTGAAGGAGGAAAAAAGAAGAAAAAAAAAAA

**cDNA sequence (742 bp)**

GAAAAAAGCAAAAGCAGTGCTCTTCCATCAGTGCTATCATTCATACATTTGTTTCCTTTACTTTTGCTATTTGCGAAGTTTCATTCCGTACATGGCTGAAGAGCAACACCATGATGCGTGCCAGGAGAGTGAGAACGCAGGAGGTGAAATCAAGGACCGTGGGATGTTTGATTTTTTGAGTAAGAAGAAGGAAGAAAAGCCTCAGGAGGAGGCTGTAGTGGCGGAGTTTGAGAAGGTACATGTGTCAGAACCCTATAAGAAGGAGGAGGACGAAAAAGAGGAGAAGCCCATTCACGCTGAGAAGATACAACGGTCAAATAGTAGCAGCTCTAGCTCGTCCAGCGATGAAGAAGAAGGTGAAGGAGGAGAAAAGAAGAAGAAGAAGAAGAAGGGATCATTGAAGGAGAAGTTATTGGGCGAGAAGAAGGAAGAAGAGAAGAAGGTGGCAGAAACGGAGGAGTGTAGAATTGGAGAAGCCACGGAGGAAAAGAAGGGATTCTTGGAGAAGATCAAAGAGAAGTTGCCTGGGCCCCCGCCCAAGAAGGTGGATGATAGTCACAACAGCACGGCGGCCGCCGCCGAGTGCAGCGCCGAACCACATGGGCACCATGAAGTGGAGGCAGCTAAGGAGAAGAAGGGTCTGTTGGAGAAGATAAAGGAGAAACTGCCTGGCTACCACAAGAATGAAGAGAAGGAGGAGTCTGCCAACCACTGAAGGAGGAAAAAAGAAGAAAAAAAAAAA

**2 5’ RACE, 3’ RACE and cDNA sequences of *LcDHN3* gene**

**5’ RACE sequence (332bp)**

GAAAACGTAAGCGAAGCATCCATAGCAAAAGCAAAAGCAAAAGCTCTTCGTTGCAATTCCCATTTCTCTCGTATTACTTTCAGGCCATGGCTGACGAGCAACACCACACCGGTTTTAGCGACGAAGAAGAAGAAGAAGAATGTGAAGCTGATGGTGAGAAGAAAAAGAAAAAGAAAGGGTTGAAGGAGAAGATCAAGGAGAAGATATCTGGCGAGAAGGAGGAAGAAG

**3’ RACE sequence (456bp)**

ATCAAGGAGAAGATATCTGGCGAGAAGGAGGAAGAAGTGAAAGTGGCTGAGTTCGAAGACACATCAATTCCCATCGAGAAGATTGAAGCAACACATGAAGATGAAGCTGCAGGAGAGAAGAAAGGCTTTCTGGAGAAGATGAAAGAAAAGCTCCCGGGAGGTCACCCAAAGAAGCCTGACGACAGTGAAGTGGTCGAGTGTGGAGATGCTGCTTCGGGGCATGAAGTTGAGGCCGGTAAGGAGAAGAAGGGACTGTTGGAGAAGATCAAAGAGAAGCTGCCTGGTTACCACAAGAATGGAGGAGAAGAGAAGGAGAGTGCCAACAAGTACTAGAGGAGTTTTGTTTTGGTAGTTCTATGGTAGGATGTGATTTCTTTTATGTAATGTGGTGATGATTTGTGTCCTTTTGATTGATGGTGATGATGATTTCATGTCTTTTATGTCAAAAAAAAAAAA

**cDNA sequence (809 bp)**

GAAAACGTAAGCGAAGCATCCATAGCAAAAGCAAAAGCAAAAGCTCTTCGTTGCAATTCCCATTTCTCTCGTATTACTTTCAGGCCATGGCTGACGAGCAACACCACACCGGTGTATGTGGGACTCATGAAAGCGAGGGTGAAATCAAAGATCGAGGAATACTCGAGCTTTTGGGTAAGAAGGAAGGAGAGGAGGTTGTGGTAACTGAGGTTGAGAAGGTCCACGTTTCAGAGCATTGTGAGAAACTCCATCGATCTCACAGCTCTTCTAGCTCGTCTAGCGACGAAGAAGAAGAAGAAGAGTGTGAAGCTGATGGTGAGAAGAAAAAGAAAAAGAAAGGGTTGAAGGAGAAGATCAAGGAGAAGATATCTGGCGAGAAGGAGGAAGAAGTGAAAGTGGCTGAGTTCGAAGACACATCAATTCCCATCGAGAAGATTGAAGCAACACATGAAGATGAAGCTGCAGGAGAGAAGAAAGGCTTTCTGGAGAAGATGAAAGAAAAGCTCCCGGGAGGTCACCCAAAGAAGCCTGACGACAGTGAAGTGGTCGAGTGTGGAGATGCTGCTTCGGGGCATGAAGTTGAGGCCGGTAAGGAGAAGAAGGGACTGTTGGAGAAGATCAAAGAGAAGCTGCCTGGTTACCACAAGAATGGAGGAGAAGAGAAGGAGAGTGCCAACAAGTACTAGAGGAGTTTTGTTTTGGTAGTTCTATGGTAGGATGTGATTTCTTTTATGTAATGTGGTGATGATTTGTGTCCTTTTGATTGATGGTGATGATGATTTCATGTCTTTTATGTCAAAAAAAAAAAA

**3 Intermediate, 5’ RACE, 3’ RACE and cDNA sequences of *LtTLP11* gene**

**intermediate sequence (802 bp)**

CGCCCGCTCATTTTAACCGTAGTCAACAACTGCCCTTTCACCGTCTGGCCCGCAATACAGCCCAACGCGGGCCACGATGTCCTCGAGCGCGGTGGCTTCGCCCTCGAAACCCTAACCCACAAATCATTCCCAGCTCCAGACCACCACTGGACCGGCCGGCTCTGGGCCCGGACCGGCTGCACTTACCACGCCGGCCGCTTCTCCCGCGCCACCGGCGACTGCGGCGGCCGCCTCGAGTGCAACGGCGCGGGCGGGAAGACACCCGCCACCCTAGCGCAGTTTAGCCTCCACCACGCCCACAACGATCAGTCCTCCTACTCTGTCAGCCTCGTGGACGGTTACAACCTTCCGATGACGGTGACCCCACACGAGGGCCAGGGCATGTGTCCCGTCGTTGGATGTAAGGCCGATCTGATTCCCACGTGTCCCCCGGCGCTACAGATGCGGGTCCCAGCCGGTCACGGGCCGGTGATGGCTTGCAAGAGCGGGTGCGAGGCGTTCGGTACGGACGAGCTGTGCTGCAGGAACCATTTCAACAGCCCGCAGACGTGCAGGGGGTCGAGCTACTCGGAGTTTTTCAAGCATGCTTGCCCGGCAACGTACACCTATGCGCACGATAGCCCCTCGCTCACCCACAACTGCGTGGCGCCGCGGGAGCTGAAGGTTATCTTTTGCCACTAATATTTAAGTGAAATTACGTGGTATGCCACTGAGAAGTGTACTAAACTAGCTCCTTAGTAGTAGGCTACCATCTGCCCTTTGTTTTTGGTTTGGTGGTTTGTGGTGCATGGATTGTCAGATC

**5’ RACE sequence (470 bp)**

CTAATACGACTCACTATAGGGCAAGCAGTGGTATCAACGCAGAGTACATGGGGAACGCTCCAACCATGGCTTGTCTCTCTTACGTTCTCCTCCCTCTGGCTGTCCTCCTTATCCTTCACCCCCTCGCCGAGTCTACGCACCGCCCGCTCATTTTAACCGTAGTCAACAACTGCCCTTTCACCGTCTGGCCCGCAATACAGCCCAACGCGGGCCACGATGTCCTCGAGCGCGGTGGCTTCGCCCTCGAAACCCTAACCCACAAATCCTTCCCAGCTCCAGACCACCACTGGACCGGCCGGCTCTGGGCCAGGACCGGCTGTACTTACCACGCCGGCCGCTTCTCCTGCGCCACCGGCGACTGCGGCGGCCGCCTCGAGTGCAACGGTGCGGGCGGGAAGACGCCCGCCACCCTAGCGCAGTTTAGCCTCCACCACGCCCACAACGACCAGTCCTCCTACTCTGTCAGCCTC

**3’ RACE sequence (544 bp)**

CCTACTCTGTCAGCCTCGTGGACGGTTACAACCTTCCGATGACGGTGACCCCACACGAGGGCCAGGGCATGTGTCCCGTCGTTGGATGTAAGGCCGATCTGATTCCCACGTGTCCCCCGGCGCTACAGATGCGGGTCCCAGCCGGTCACGGGCCGGTGATGGCTTGCAAGAGCGGGTGCGAGGCGTTCGGTACGGACGAGCTGTGCTGCAGGAACCATTTCAACAGCCCGCAGACGTGCAGGGGGTCGAGCTACTCGGAGTTTTTCAAGCATGCTTGCCCGGCAACGTACACCTATGCGCACGATAGCCCCTCGCTCACCCACAACTGCGTGGCGCCGCGGGAGCTGAAGGTTATCTTTTGCCACTAATATTTAAGTGAAATTACGTGGTATGCCACTGAGAAGTGTACTAAACTAGCTCCTTAGTAGTAGGCTACCATCTGCCCTTTGTTTTTGGTTTGGTGGTTTGTGGTGCATGGATTGTCAGATCATGTGTACGTTCTCTATAAGACTATAACGTGTGTTATTTTTATAAAAAAAAAAAA

**cDNA sequence (997 bp)**

CTAATACGACTCACTATAGGGCAAGCAGTGGTATCAACGCAGAGTACATGGGGAACGCTCCAACCATGGCTTGTCTCTCTTACGTTCTCCTCCCTCTGGCTGTCCTCCTTATCCTTCACCCCCTCGCCGAGTCTACGCACCGCCCGCTCATTTTAACCGTAGTCAACAACTGCCCTTTCACCGTCTGGCCCGCAATACAGCCCAACGCGGGCCACGATGTCCTCGAGCGCGGTGGCTTCGCCCTCGAAACCCTAACCCACAAATCCTTCCCAGCTCCAGACCACCACTGGACCGGCCGGCTCTGGGCCAGGACCGGCTGTACTTACCACGCCGGCCGCTTCTCCTGCGCCACCGGCGACTGCGGCGGCCGCCTCGAGTGCAACGGTGCGGGCGGGAAGACGCCCGCCACCCTAGCGCAGTTTAGCCTCCACCACGCCCACAACGACCAGTCCTCCTACTCTGTCAGCCTCGTGGACGGTTACAACCTTCCGATGACGGTGACCCCACACGAGGGCCAGGGCATGTGTCCCGTCGTTGGATGTAAGGCCGATCTGATTCCCACGTGTCCCCCGGCGCTACAGATGCGGGTCCCAGCCGGTCACGGGCCGGTGATGGCTTGCAAGAGCGGGTGCGAGGCGTTCGGTACGGACGAGCTGTGCTGCAGGAACCATTTCAACAGCCCGCAGACGTGCAGGGGGTCGAGCTACTCGGAGTTTTTCAAGCATGCTTGCCCGGCAACGTACACCTATGCGCACGATAGCCCCTCGCTCACCCACAACTGCGTGGCGCCGCGGGAGCTGAAGGTTATCTTTTGCCACTAATATTTAAGTGAAATTACGTGGTATGCCACTGAGAAGTGTACTAAACTAGCTCCTTAGTAGTAGGCTACCATCTGCCCTTTGTTTTTGGTTTGGTGGTTTGTGGTGCATGGATTGTCAGATCATGTGTACGTTCTCTATAAGACTATAACGTGTGTTATTTTTATAAAAAAAAAAAA
