# Supplementary material for: Population Genetic Differentiation and Evolutionary History in Liriodendron Revealed by Stress‐Related Single‐Copy Genes
Source: Ecol Evol. 2025 Sep 22;15(9):e72182. doi: 10.1002/ece3.72182 (PMC12453614; doi:10.1002/ece3.72182)
Supplement: Supplementary file 2 — Appendix B. [file ECE3-15-e72182-s003.docx]

**RNA Sequence and Amino Acid Translation of *LtDHN2*, *LtDHN3* and *LtTLP11* gene**

**1. *LtDHN2***

　１　ＡＴＧＧＣＴＧＡＡＧＡＧＣＡＡＣＡＣＣＡＴＧＡＴＧＣＧＴＧＣＣＡＧＧＡＧＡＧＴＧＡＧＡＡＣ

　＊　Ｍ　　Ａ　　Ｅ　　Ｅ　　Ｑ　　Ｈ　　Ｈ　　Ｄ　　Ａ　　Ｃ　　Ｑ　　Ｅ　　Ｓ　　Ｅ　　Ｎ　15

　４６ＧＣＡＧＧＡＧＧＴＧＡＡＡＴＣＡＡＧＧＡＣＣＧＴＧＧＧＡＴＧＴＴＴＧＡＴＴＴＴＴＴＧＡＧＴ

　　　Ａ　　Ｇ　　Ｇ　　Ｅ　　Ｉ　　Ｋ　　Ｄ　　Ｒ　　Ｇ　　Ｍ　　Ｆ　　Ｄ　　Ｆ　　Ｌ　　Ｓ　30

　９１ＡＡＧＡＡＧＡＡＧＧＡＡＧＡＡＡＡＧＣＣＴＣＡＧＧＡＧＧＡＧＧＣＴＧＴＡＧＴＧＧＣＧＧＡＧ

　　　Ｋ　　Ｋ　　Ｋ　　Ｅ　　Ｅ　　Ｋ　　Ｐ　　Ｑ　　Ｅ　　Ｅ　　Ａ　　Ｖ　　Ｖ　　Ａ　　Ｅ　45

１３６ＴＴＴＧＡＧＡＡＧＧＴＡＣＡＴＧＴＧＴＣＡＧＡＡＣＣＣＴＡＴＡＡＧＡＡＧＧＡＧＧＡＧＧＡＣ

　　　Ｆ　　Ｅ　　Ｋ　　Ｖ　　Ｈ　　Ｖ　　Ｓ　　Ｅ　　Ｐ　　Ｙ　　Ｋ　　Ｋ　　Ｅ　　Ｅ　　Ｄ　60

１８１ＧＡＡＡＡＡＧＡＧＧＡＧＡＡＧＣＣＣＡＴＴＣＡＣＧＣＴＧＡＧＡＡＧＡＴＡＣＡＡＣＧＧＴＣＡ

　　　Ｅ　　Ｋ　　Ｅ　　Ｅ　　Ｋ　　Ｐ　　Ｉ　　Ｈ　　Ａ　　Ｅ　　Ｋ　　Ｉ　　Ｑ　　Ｒ　　Ｓ　75

２２６ＡＡＴＡＧＴＡＧＣＡＧＣＴＣＴＡＧＣＴＣＧＴＣＣＡＧＣＧＡＴＧＡＡＧＡＡＧＡＡＧＧＴＧＡＡ

　　　Ｎ　　Ｓ　　Ｓ　　Ｓ　　Ｓ　　Ｓ　　Ｓ　　Ｓ　　Ｓ　　Ｄ　　Ｅ　　Ｅ　　Ｅ　　Ｇ　　Ｅ　90

２７１ＧＧＡＧＧＡＧＡＡＡＡＧＡＡＧＡＡＧＡＡＧＡＡＧＡＡＧＡＡＧＧＧＡＴＣＡＴＴＧＡＡＧＧＡＧ

　　　Ｇ　　Ｇ　　Ｅ　　Ｋ　　Ｋ　　Ｋ　　Ｋ　　Ｋ　　Ｋ　　Ｋ　　Ｇ　　Ｓ　　Ｌ　　Ｋ　　Ｅ　105

３１６ＡＡＧＴＴＡＴＴＧＧＧＣＧＡＧＡＡＧＡＡＧＧＡＡＧＡＡＧＡＧＡＡＧＡＡＧＧＴＧＧＣＡＧＡＡ

　　　Ｋ　　Ｌ　　Ｌ　　Ｇ　　Ｅ　　Ｋ　　Ｋ　　Ｅ　　Ｅ　　Ｅ　　Ｋ　　Ｋ　　Ｖ　　Ａ　　Ｅ　120

３６１ＡＣＧＧＡＧＧＡＧＴＧＴＡＧＡＡＴＴＧＧＡＧＡＡＧＣＣＡＣＧＧＡＧＧＡＡＡＡＧＡＡＧＧＧＡ

Ｔ　　Ｅ　　Ｅ　　Ｃ　　Ｒ　　Ｉ　　Ｇ　　Ｅ　　Ａ　　Ｔ　　Ｅ　　Ｅ　　Ｋ　　Ｋ　　Ｇ　135

４０６ＴＴＣＴＴＧＧＡＧＡＡＧＡＴＣＡＡＡＧＡＧＡＡＧＴＴＧＣＣＴＧＧＧＣＣＣＣＣＧＣＣＣＡＡＧ

　Ｆ　　Ｌ　　Ｅ　　Ｋ　　Ｉ　　Ｋ　　Ｅ　　Ｋ　　Ｌ　　Ｐ　　Ｇ　　Ｐ　　Ｐ　　Ｐ　　Ｋ　150

４５１ＡＡＧＧＴＧＧＡＴＧＡＴＡＧＴＣＡＣＡＡＣＡＧＣＡＣＧＧＣＧＧＣＣＧＣＣＧＣＣＧＡＧＴＧＣ

　　　Ｋ　　Ｖ　　Ｄ　　Ｄ　　Ｓ　　Ｈ　　Ｎ　　Ｓ　　Ｔ　　Ａ　　Ａ　　Ａ　　Ａ　　Ｅ　　Ｃ　165

４９６ＡＧＣＧＣＣＧＡＡＣＣＡＣＡＴＧＧＧＣＡＣＣＡＴＧＡＡＧＴＧＧＡＧＧＣＡＧＣＴＡＡＧＧＡＧ

　　　Ｓ　　Ａ　　Ｅ　　Ｐ　　Ｈ　　Ｇ　　Ｈ　　Ｈ　　Ｅ　　Ｖ　　Ｅ　　Ａ　　Ａ　　Ｋ　　Ｅ　180

５４１ＡＡＧＡＡＧＧＧＴＣＴＧＴＴＧＧＡＧＡＡＧＡＴＡＡＡＧＧＡＧＡＡＡＣＴＧＣＣＴＧＧＣＴＡＣ

　　　Ｋ　　Ｋ　　Ｇ　　Ｌ　　Ｌ　　Ｅ　　Ｋ　　Ｉ　　Ｋ　　Ｅ　　Ｋ　　Ｌ　　Ｐ　　Ｇ　　Ｙ　195

５８６ＣＡＣＡＡＧＡＡＴＧＡＡＧＡＧＡＡＧＧＡＧＧＡＧＴＣＴＧＣＣＡＡＣＣＡＣＴＧＡ

　　　Ｈ　　Ｋ　　Ｎ　　Ｅ　　Ｅ　　Ｋ　　Ｅ　　Ｅ　　Ｓ　　Ａ　　Ｎ　　Ｈ　　＊＊　　　　　　207

**2. *LtDHN3***

　１　ＡＡＡＡＧＣＡＡＡ

　１０ＡＧＣＴＣＴＴＣＧＴＴＧＣＡＡＴＴＣＣＣＡＴＴＴＣＴＣＴＣＧＴＡＴＴＡＣＴＴＴＣＡＧＧＣＣ

　５５ＡＴＧＧＣＴＧＡＣＧＡＧＣＡＡＣＡＣＣＡＣＡＣＣＧＧＴＧＴＡＴＧＴＧＧＧＡＣＴＣＡＴＧＡＡ

　　　Ｍ　　Ａ　　Ｄ　　Ｅ　　Ｑ　　Ｈ　　Ｈ　　Ｔ　　Ｇ　　Ｖ　　Ｃ　　Ｇ　　Ｔ　　Ｈ　　Ｅ　１５

１００ＡＧＣＧＡＧＧＧＴＧＡＡＡＴＣＡＡＡＧＡＴＣＧＡＧＧＡＡＴＡＣＴＣＧＡＧＣＴＴＴＴＧＧＧＴ

　　　Ｓ　　Ｅ　　Ｇ　　Ｅ　　Ｉ　　Ｋ　　Ｄ　　Ｒ　　Ｇ　　Ｉ　　Ｌ　　Ｅ　　Ｌ　　Ｌ　　Ｇ　３０

１４５ＡＡＧＡＡＧＧＡＡＧＧＡＧＡＧＧＡＧＧＴＴＧＴＧＧＴＡＡＣＴＧＡＧＧＴＴＧＡＧＡＡＧＧＴＣ

　　　Ｋ　　Ｋ　　Ｅ　　Ｇ　　Ｅ　　Ｅ　　Ｖ　　Ｖ　　Ｖ　　Ｔ　　Ｅ　　Ｖ　　Ｅ　　Ｋ　　Ｖ　４５

１９０ＣＡＣＧＴＴＴＣＡＧＡＧＣＡＴＴＧＴＧＡＧＡＡＡＣＴＣＣＡＴＣＧＡＴＣＴＣＡＣＡＧＣＴＣＴ

　　　Ｈ　　Ｖ　　Ｓ　　Ｅ　　Ｈ　　Ｃ　　Ｅ　　Ｋ　　Ｌ　　Ｈ　　Ｒ　　Ｓ　　Ｈ　　Ｓ　　Ｓ　６０

２３５ＴＣＴＡＧＣＴＣＧＴＣＴＡＧＣＧＡＣＧＡＡＧＡＡＧＡＡＧＡＡＧＡＡＧＡＧＴＧＴＧＡＡＧＣＴ

　　　Ｓ　　Ｓ　　Ｓ　　Ｓ　　Ｓ　　Ｄ　　Ｅ　　Ｅ　　Ｅ　　Ｅ　　Ｅ　　Ｅ　　Ｃ　　Ｅ　　Ａ　７５

２８０ＧＡＴＧＧＴＧＡＧＡＡＧＡＡＡＡＡＧＡＡＡＡＡＧＡＡＡＧＧＧＴＴＧＡＡＧＧＡＧＡＡＧＡＴＣ

　　　Ｄ　　Ｇ　　Ｅ　　Ｋ　　Ｋ　　Ｋ　　Ｋ　　Ｋ　　Ｋ　　Ｇ　　Ｌ　　Ｋ　　Ｅ　　Ｋ　　Ｉ　９０

３２５ＡＡＧＧＡＧＡＡＧＡＴＡＴＣＴＧＧＣＧＡＧＡＡＧＧＡＧＧＡＡＧＡＡＧＴＧＡＡＡＧＴＧＧＣＴ

　　　Ｋ　　Ｅ　　Ｋ　　Ｉ　　Ｓ　　Ｇ　　Ｅ　　Ｋ　　Ｅ　　Ｅ　　Ｅ　　Ｖ　　Ｋ　　Ｖ　　Ａ　１０５

３７０ＧＡＧＴＴＣＧＡＡＧＡＣＡＣＡＴＣＡＡＴＴＣＣＣＡＴＣＧＡＧＡＡＧＡＴＴＧＡＡＧＣＡＡＣＡ

　　　Ｅ　　Ｆ　　Ｅ　　Ｄ　　Ｔ　　Ｓ　　Ｉ　　Ｐ　　Ｉ　　Ｅ　　Ｋ　　Ｉ　　Ｅ　　Ａ　　Ｔ　１２０

４１５ＣＡＴＧＡＡＧＡＴＧＡＡＧＣＴＧＣＡＧＧＡＧＡＧＡＡＧＡＡＡＧＧＣＴＴＴＣＴＧＧＡＧＡＡＧ

　　　Ｈ　　Ｅ　　Ｄ　　Ｅ　　Ａ　　Ａ　　Ｇ　　Ｅ　　Ｋ　　Ｋ　　Ｇ　　Ｆ　　Ｌ　　Ｅ　　Ｋ　１２５

４６０ＡＴＧＡＡＡＧＡＡＡＡＧＣＴＣＣＣＧＧＧＡＧＧＴＣＡＣＣＣＡＡＡＧＡＡＧＣＣＴＧＡＣＧＡＣ

　　　Ｍ　　Ｋ　　Ｅ　　Ｋ　　Ｌ　　Ｐ　　Ｇ　　Ｇ　　Ｈ　　Ｐ　　Ｋ　　Ｋ　　Ｐ　　Ｄ　　Ｄ　１５０

５０５ＡＧＴＧＡＡＧＴＧＧＴＣＧＡＧＴＧＴＧＧＡＧＡＴＧＣＴＧＣＴＴＣＧＧＧＧＣＡＴＧＡＡＧＴＴ

　　　Ｓ　　Ｅ　　Ｖ　　Ｖ　　Ｅ　　Ｃ　　Ｇ　　Ｄ　　Ａ　　Ａ　　Ｓ　　Ｇ　　Ｈ　　Ｅ　　Ｖ　１６５

５５０ＧＡＧＧＣＣＧＧＴＡＡＧＧＡＧＡＡＧＡＡＧＧＧＡＣＴＧＴＴＧＧＡＧＡＡＧＡＴＣＡＡＡＧＡＧ

　　　Ｅ　　Ａ　　Ｇ　　Ｋ　　Ｅ　　Ｋ　　Ｋ　　Ｇ　　Ｌ　　Ｌ　　Ｅ　　Ｋ　　Ｉ　　Ｋ　　Ｅ　１８０

５９５ＡＡＧＣＴＧＣＣＴＧＧＴＴＡＣＣＡＣＡＡＧＡＡＴＧＧＡＧＧＡＧＡＡＧＡＧＡＡＧＧＡＧＡＧＴ

　　　Ｋ　　Ｌ　　Ｐ　　Ｇ　　Ｙ　　Ｈ　　Ｋ　　Ｎ　　Ｇ　　Ｇ　　Ｅ　　Ｅ　　Ｋ　　Ｅ　　Ｓ　１９５

６４０ＧＣＣＡＡＣＡＡＧＴＡＣＴＡＧ

　　　Ａ　　Ｎ　　Ｋ　　Ｙ　　＊＊　　　　　　　　　　　　　　　　　　　　　　　　　　　　　　２１０

７３０ＡＧＧＡＧＴＴＴＴＧＴＴＴＴＧＧＴＡＧＴＴＣＴＡＴＧＧＴＡＧＧＡＴＧＴＧＡＴＴＴＣＴＴＴＴ

７７５ＡＴＧＴＡＡＴＧＴＧＧＴＧＡＴＧＡＴＴＴＧＴＧＴＣＣＴＴＴＴＧＡＴＴＧＡＴＧ

**3. *LtTLP11***

　　１ＡＴＧＧＧＧＡＡＣＧＣＴＣＣＡＡＣＣＡＴＧＧＣＴＴＧＴＣＴＣＴＣＴＴＡＣＧＴＴＣＴＣＣＴＣ

　＊　Ｍ　　Ｇ　　Ｎ　　Ａ　　Ｐ　　Ｔ　　Ｍ　　Ａ　　Ｃ　　Ｌ　　Ｓ　　Ｙ　　Ｖ　　Ｌ　　Ｌ　15

　４６ＣＣＴＣＴＧＧＣＴＧＴＣＣＴＣＣＴＴＡＴＣＣＴＴＣＡＣＣＣＣＣＴＣＧＣＣＧＡＧＴＣＴＡＣＧ

　　　Ｐ　　Ｌ　　Ａ　　Ｖ　　Ｌ　　Ｌ　　Ｉ　　Ｌ　　Ｈ　　Ｐ　　Ｌ　　Ａ　　Ｅ　　Ｓ　　Ｔ　30

　９１ＣＡＣＣＧＣＣＣＧＣＴＣＡＴＴＴＴＡＡＣＣＧＴＡＧＴＣＡＡＣＡＡＣＴＧＣＣＣＴＴＴＣＡＣＣ

　　　Ｈ　　Ｒ　　Ｐ　　Ｌ　　Ｉ　　Ｌ　　Ｔ　　Ｖ　　Ｖ　　Ｎ　　Ｎ　　Ｃ　　Ｐ　　Ｆ　　Ｔ　45

１３６ＧＴＣＴＧＧＣＣＣＧＣＡＡＴＡＣＡＧＣＣＣＡＡＣＧＣＧＧＧＣＣＡＣＧＡＴＧＴＣＣＴＣＧＡＧ

　　　Ｖ　　Ｗ　　Ｐ　　Ａ　　Ｉ　　Ｑ　　Ｐ　　Ｎ　　Ａ　　Ｇ　　Ｈ　　Ｄ　　Ｖ　　Ｌ　　Ｅ　60

１８１ＣＧＣＧＧＴＧＧＣＴＴＣＧＣＣＣＴＣＧＡＡＡＣＣＣＴＡＡＣＣＣＡＣＡＡＡＴＣＣＴＴＣＣＣＡ

　　　Ｒ　　Ｇ　　Ｇ　　Ｆ　　Ａ　　Ｌ　　Ｅ　　Ｔ　　Ｌ　　Ｔ　　Ｈ　　Ｋ　　Ｓ　　Ｆ　　Ｐ　75

２２６ＧＣＴＣＣＡＧＡＣＣＡＣＣＡＣＴＧＧＡＣＣＧＧＣＣＧＧＣＴＣＴＧＧＧＣＣＡＧＧＡＣＣＧＧＣ

　　　Ａ　　Ｐ　　Ｄ　　Ｈ　　Ｈ　　Ｗ　　Ｔ　　Ｇ　　Ｒ　　Ｌ　　Ｗ　　Ａ　　Ｒ　　Ｔ　　Ｇ　90

２７１ＴＧＴＡＣＴＴＡＣＣＡＣＧＣＣＧＧＣＣＧＣＴＴＣＴＣＣＴＧＣＧＣＣＡＣＣＧＧＣＧＡＣＴＧＣ

　　　Ｃ　　Ｔ　　Ｙ　　Ｈ　　Ａ　　Ｇ　　Ｒ　　Ｆ　　Ｓ　　Ｃ　　Ａ　　Ｔ　　Ｇ　　Ｄ　　Ｃ　105

３１６ＧＧＣＧＧＣＣＧＣＣＴＣＧＡＧＴＧＣＡＡＣＧＧＴＧＣＧＧＧＣＧＧＧＡＡＧＡＣＧＣＣＣＧＣＣ

　　　Ｇ　　Ｇ　　Ｒ　　Ｌ　　Ｅ　　Ｃ　　Ｎ　　Ｇ　　Ａ　　Ｇ　　Ｇ　　Ｋ　　Ｔ　　Ｐ　　Ａ　120

３６１ＡＣＣＣＴＡＧＣＧＣＡＧＴＴＴＡＧＣＣＴＣＣＡＣＣＡＣＧＣＣＣＡＣＡＡＣＧＡＣＣＡＧＴＣＣ

　　　Ｔ　　Ｌ　　Ａ　　Ｑ　　Ｆ　　Ｓ　　Ｌ　　Ｈ　　Ｈ　　Ａ　　Ｈ　　Ｎ　　Ｄ　　Ｑ　　Ｓ　135

４０６ＴＣＣＴＡＣＴＣＴＧＴＣＡＧＣＣＴＣＧＴＧＧＡＣＧＧＴＴＡＣＡＡＣＣＴＴＣＣＧＡＴＧＡＣＧ

　　　Ｓ　　Ｙ　　Ｓ　　Ｖ　　Ｓ　　Ｌ　　Ｖ　　Ｄ　　Ｇ　　Ｙ　　Ｎ　　Ｌ　　Ｐ　　Ｍ　　Ｔ　150

４５１ＧＴＧＡＣＣＣＣＡＣＡＣＧＡＧＧＧＣＣＡＧＧＧＣＡＴＧＴＧＴＣＣＣＧＴＣＧＴＴＧＧＡＴＧＴ

　　　Ｖ　　Ｔ　　Ｐ　　Ｈ　　Ｅ　　Ｇ　　Ｑ　　Ｇ　　Ｍ　　Ｃ　　Ｐ　　Ｖ　　Ｖ　　Ｇ　　Ｃ　165

４９６ＡＡＧＧＣＣＧＡＴＣＴＧＡＴＴＣＣＣＡＣＧＴＧＴＣＣＣＣＣＧＧＣＧＣＴＡＣＡＧＡＴＧＣＧＧ

　　　Ｋ　　Ａ　　Ｄ　　Ｌ　　Ｉ　　Ｐ　　Ｔ　　Ｃ　　Ｐ　　Ｐ　　Ａ　　Ｌ　　Ｑ　　Ｍ　　Ｒ　180

５４１ＧＴＣＣＣＡＧＣＣＧＧＴＣＡＣＧＧＧＣＣＧＧＴＧＡＴＧＧＣＴＴＧＣＡＡＧＡＧＣＧＧＧＴＧＣ

　　　Ｖ　　Ｐ　　Ａ　　Ｇ　　Ｈ　　Ｇ　　Ｐ　　Ｖ　　Ｍ　　Ａ　　Ｃ　　Ｋ　　Ｓ　　Ｇ　　Ｃ　195

５８６ＧＡＧＧＣＧＴＴＣＧＧＴＡＣＧＧＡＣＧＡＧＣＴＧＴＧＣＴＧＣＡＧＧＡＡＣＣＡＴＴＴＣＡＡＣ

　　　Ｅ　　Ａ　　Ｆ　　Ｇ　　Ｔ　　Ｄ　　Ｅ　　Ｌ　　Ｃ　　Ｃ　　Ｒ　　Ｎ　　Ｈ　　Ｆ　　Ｎ　210

６３１ＡＧＣＣＣＧＣＡＧＡＣＧＴＧＣＡＧＧＧＧＧＴＣＧＡＧＣＴＡＣＴＣＧＧＡＧＴＴＴＴＴＣＡＡＧ

　　　Ｓ　　Ｐ　　Ｑ　　Ｔ　　Ｃ　　Ｒ　　Ｇ　　Ｓ　　Ｓ　　Ｙ　　Ｓ　　Ｅ　　Ｆ　　Ｆ　　Ｋ　225

６７６ＣＡＴＧＣＴＴＧＣＣＣＧＧＣＡＡＣＧＴＡＣＡＣＣＴＡＴＧＣＧＣＡＣＧＡＴＡＧＣＣＣＣＴＣＧ

　　　Ｈ　　Ａ　　Ｃ　　Ｐ　　Ａ　　Ｔ　　Ｙ　　Ｔ　　Ｙ　　Ａ　　Ｈ　　Ｄ　　Ｓ　　Ｐ　　Ｓ　240

７２１ＣＴＣＡＣＣＣＡＣＡＡＣＴＧＣＧＴＧＧＣＧＣＣＧＣＧＧＧＡＧＣＴＧＡＡＧＧＴＴＡＴＣＴＴＴ

　　　Ｌ　　Ｔ　　Ｈ　　Ｎ　　Ｃ　　Ｖ　　Ａ　　Ｐ　　Ｒ　　Ｅ　　Ｌ　　Ｋ　　Ｖ　　Ｉ　　Ｆ　255

７６６ＴＧＣＣＡＣＴＡＡ

　　　Ｃ　　Ｈ　　＊＊　　　　　　　　　　　　　　　　　　　　　　　　　　　　　　　　　　　　257
