# Supplementary material for: Population Genetic Differentiation and Evolutionary History in Liriodendron Revealed by Stress‐Related Single‐Copy Genes
Source: Ecol Evol. 2025 Sep 22;15(9):e72182. doi: 10.1002/ece3.72182 (PMC12453614; doi:10.1002/ece3.72182)
Supplement: Supplementary file 3 — Table S1: ece372182‐sup‐0003‐TableS1.docx. [file ECE3-15-e72182-s002.docx]

**Table S1 Primers for *LtDHN2*, *LtDHN3*, *LtTLP11* cloning**

| **基因**  **Genes** | **引物类型**  **The type of primers** | **引物名称**  **Primers name** | **引物序列**  **Primers sequences（5'→3'）** | **退火温度**  **Tm/℃** |
| --- | --- | --- | --- | --- |
| ***LtDHN2*** | Primers of 3′RACE | *LtDHN23F1* | ACAACGGTCAAATAGTAGCAGCTCT | 61℃ |
|  |  | *LtDHN23F2* | TTGAAGGAGAAGTTATTGGGCGAGA | 60℃ |
|  | Primers of 5′RACE | *LtDHN25R1* | TTCTTCTCCTTAGCTGCCTCCAC | 60℃ |
|  |  | *LtDHN25R2* | ATTCTACACTCCTCCGTTTCTGC | 60℃ |
|  | Primers of ORF | *LtDHN2OF* | ATGGGGAAGAAGGAAGAAAAG | 50℃ |
|  |  | *LtDHN2OR* | TCAGTGGTTGGCAGACTC |  |
| ***LtDHN3*** | Primers of 3′RACE | *LtDHN33F1* | TGAAATCAAAGATCGAGGAA | 47℃ |
|  |  | *LtDHN33F2* | ATCAAGGAGAAGATATCTGGC | 50℃ |
|  | Primers of 5′RACE | *LtDHN35R1* | AGTACTTGTTGGCACTCTCC | 52℃ |
|  |  | *LtDHN35R2* | CTTCTTCCTCCTTCTCGCCAG | 56℃ |
|  | Primers of ORF | LtDHN3OF | AAAAGCAAAAGCTCTTCG | 46℃ |
|  |  | LtDHN3OR | CATCAATCAAAAGGACACAAA |  |
| ***LtTLP11*** | Primers of intermediate fragment | *LtTLP11F* | CGCCCGCTCATTTTAACCGTA | 59℃ |
|  |  | *LtTLP11R* | GATCTGACAATCCATGCACCAC |  |
|  | Primers of 3′RACE | *LtTLP11F1* | CGCCCGCTCATTTTAACCGTA | 59℃ |
|  |  | *LtTLP11F2* | CCTACTCTGTCAGCCTCGTG | 58℃ |
|  | Primers of 5′RACE | *LtTLP11R1* | ATGATCTGACAATCCATGCACCAC | 60℃ |
|  |  | *LtTLP11R2* | GAGGCTGACAGAGTAGGAGGAC | 59℃ |
|  | Primers of ORF | *LtTLP11OF* | ATGGGGAACGCTCCAAC | 50℃ |
|  |  | *LtTLP11OR* | TTAGTGGCAAAAGATAACCTTC |  |
